# Intrinsic adriamycin resistance in p53-mutated breast cancer is related to the miR-30c/FANCF/REV1-mediated DNA damage response

**DOI:** 10.1038/s41419-019-1871-z

**Published:** 2019-09-11

**Authors:** Shu Lin, Lifeng Yu, Xinyue Song, Jia Bi, Longyang Jiang, Yan Wang, Miao He, Qinghuan Xiao, Mingli Sun, Olufunmilayo I. Olopade, Lin Zhao, Minjie Wei

**Affiliations:** 1 Department of Pharmacology, School of Pharmacy, China Medical University, No. 77 Puhe Road, Shenyang North New Area, 110122 Shenyang City, Liaoning China; 2 Liaoning Key Laboratory of molecular targeted anti-tumor drug development and evaluation, China Medical University, No.77 Puhe Road, Shenyang North New Area, 110122 Shenyang City, Liaoning China; 3 Section of Hematology/Oncology, Department of Medicine, University of Chicago, Chicago, IL 60637-1463 USA

**Keywords:** Cell biology, Cell biology, Molecular biology, Molecular biology

## Abstract

Adriamycin(ADR) is still considered to be one of the most effective agents in the treatment of breast cancer (BrCa), its efficacy is compromised by intrinsic resistance or acquire characteristics of multidrug resistance. At present, there are few genetic alterations that can be exploited as biomarkers to guide targeted use of ADR in clinical. Therefore, exploring the determinants of ADR sensitivity is pertinent for their optimal clinical application. TP53 is the most frequently mutated gene in human BrCa, p53 mutation has been reported to be closely related to ADR resistance, whereas the underlying mechanisms that cause endogenous ADR resistance in p53-mutant BrCa cells are not completely understood. The aim of the present study was to investigate the potential roles of miRNA in the response to ADR in *p53*-mutated breast cancer. Here, we report that BrCa cells expressing mutp53 are more resistant to ADR than cells with wild-type p53 (wtp53). The DNA repair protein- Fanconi anemia complementation group F protein (FANCF) and the translesion synthesis DNA polymerase REV1 protein is frequently abundant in the context of mutant p53 of BrCa. By targeting two key factors, miR-30c increases the sensitivity of BrCa cells to ADR. Furthermore, p53 directly activates the transcription of miR-30c by binding to its promoter. Subsequent analyses revealed that p53 regulates REV1 and FANCF by modulating miR-30c expression. Mutation of the p53 abolished this response. Consistently, reduced miR-30c expression is highly correlated with human BrCa with p53 mutational status and is associated with poor survival. We propose that one of the pathways affected by mutant p53 to increase intrinsic resistance to ADR involves miR-30c downregulation and the consequent upregulation of FANCF and REV1. The novel miRNA-mediated pathway that regulates chemoresistance in breast cancer will facilitate the development of novel therapeutic strategies.

## Introduction

Breast cancer is the most commonly diagnosed cancer in women and the fifth leading cause of cancer-related death worldwide, despite recent advances in therapeutic options^1^. ADR is considered one of the most effective agents for the treatment of BrCa, particularly following tamoxifen failure, but its efficacy as a curative agent is compromised by intrinsic resistance and the acquisition of multidrug resistance characteristics during chemotherapy^2^.In general, prognostic factors are helpful for better individual risk stratification and clinical outcome prediction. At present, few genetic alterations have been identified that can be exploited as biomarkers to guide the targeted use of ADR clinically. Therefore, exploring the determinants of ADR sensitivity is pertinent for their optimal clinical application.

In addition to chemotherapeutic agents, genotypic changes play a decisive role in determining the fate of cancer cells after exposure to chemotherapeutic agents. TP53 is the most frequently altered gene in human cancers^3^. Missense mutp53 protein(s) are very stable, and they acquire oncogenic properties that increase metastasis, proliferation, and cell survival^4^. p53 mutations also contribute to resistance to a variety of standard chemotherapies. A correlation between the expression of mutant p53 and resistance to ADR therapy has been observed in BrCa patients^5,6^. Identifying the signaling pathways that participate with mutp53 to promote BC chemotherapy resistance is a rational approach to unmask candidate drug targets.

ADR causes DNA damage, triggering cells to activate their DNA damage repair machinery. Deficiency in the proteins involved in DNA damage repair is considered a major determinant of the response to chemotherapy in cancer cells^7,8^. Proteome-wide analysis of mutp53 targets in BrCa identified new gain-of-function mutants, some of which involved DNA repair-related proteins^5,6,9^. However, little is known about DNA repair during the development and treatment of breast tumors with p53 mutations. Therefore, extensive investigations on the DNA repair functions that are deregulated with intrinsic ADR resistance in p53-mutated BrCa are required.

Currently, there is a growing interest in the therapeutic potential of strategies aimed to target microRNA (miRNA) networks. In the past decade, miRNAs were identified as a novel class of pivotal regulators of gene expression by base pairing with the 3′-untranslated region (UTR) of target mRNAs^10^. As each miRNA can control hundreds of gene targets, we explored the possibility that miRNAs simultaneously target deregulated DNA repair proteins in p53-mutated, ADR-resistant BrCa.

Here, we uncovered the mechanism of resistance to ADR in p53-mutated BrCa, which involves the miRNA-mediated regulation of the DNA repair protein FANCF and the translesion synthesis DNA polymerase REV1. Specifically, we identified a miRNA, miR-30c, that regulates the expression of FANCF and REV1 and specifically suppresses DNA damage repair. Consistent with these effects, overexpression of miR-30c enhanced breast cancer chemosensitivity to ADR in p53-mutant BrCa cells. Furthermore, reduced miR-30c expression was highly correlated with human BrCa p53 mutational status and was associated with poor survival, suggesting its direct clinical relevance in patients with BrCa. Our findings highlight the functions and roles of miRNAs in BrCa and suggest that resistance to ADR in p53-mutant BrCa may be related to miR-30c/FANCF/REV1-mediated DNA damage response.

## Results

### ADR resistance in p53-mutated BrCa cells is related to the high expression of FANCF and REV1

**Fig. 1:**
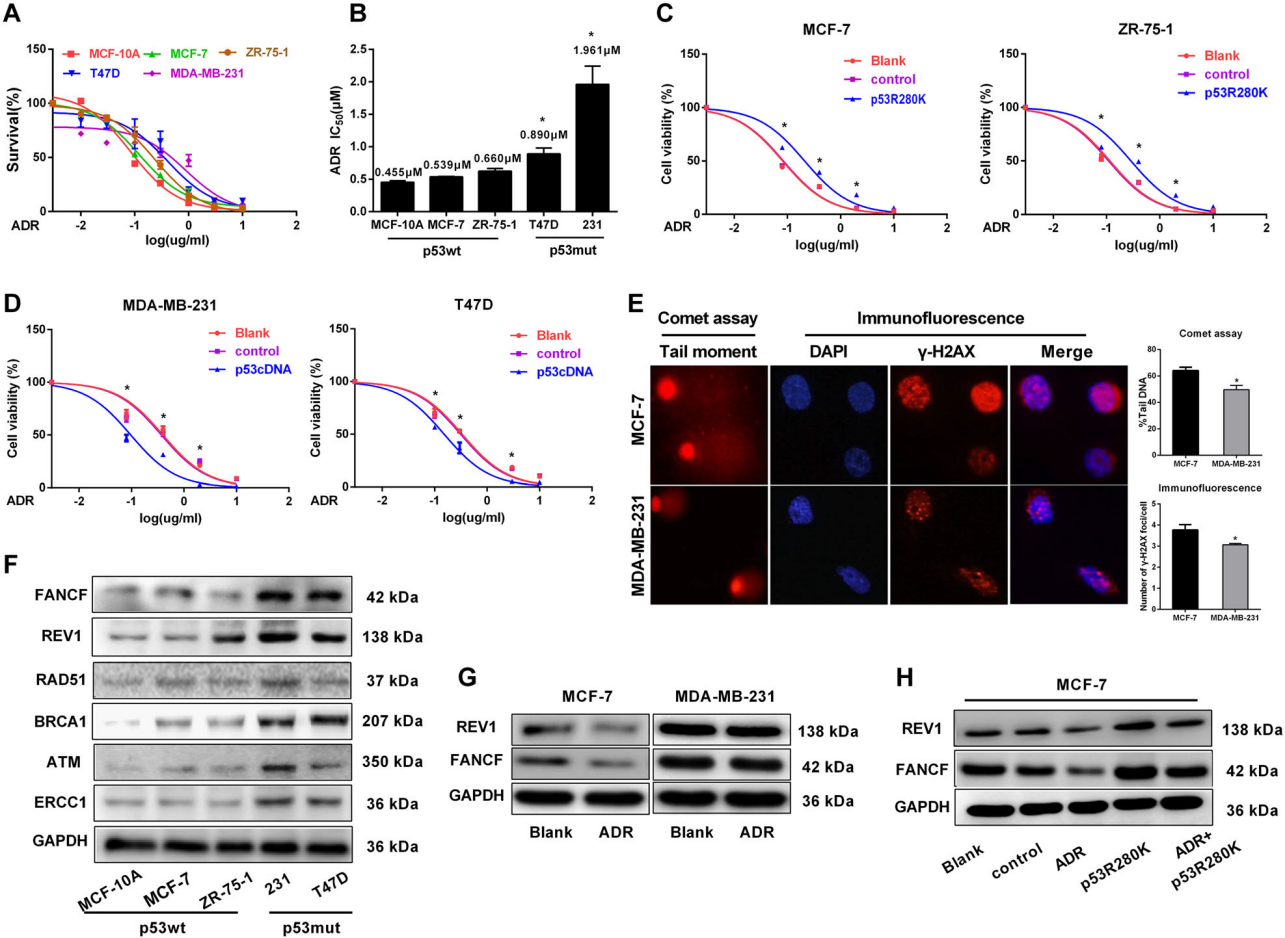
p53 status confers ADR resistance through the upregulation of FANCF and REV1 expression. **a** Determination of cell viability by Cell counting kit-8 (CCK-8) assays in the presence of various ADR concentrations with BrCa cells of known p53 status; mutP53 cells include MDA-MB-231 and T-47D, and wtp53 cells include MCF-10A, MCF-7, and ZR-75-1. **b** The IC_50_ value of BrCa cells with different p53 status. The IC_50_ value was calculated using Graphpad 7.0 software. *p < 0.05 vs. p53wt BrCa cells. **c** MCF-7 and ZR-75-1 cells expressing an empty vector (control) or transduced with mutp53 protein (p53R280K) and untreated MCF-7 and ZR-75-1 cells (blank) were treated with various ADR concentrations. Cell viability was assessed by CCK-8 assays. *p < 0.05 vs. control group. **d** MDA-MB-231 and T47D cells expressing an empty vector (control) or transduced with wtp53 protein (p53cDNA) and untreated MDA-MB-231 and T47D cells (blank) were treated with various ADR concentrations. Cell viability was assessed by CCK-8 assays. *p < 0.05 vs. control group. **e** After MCF-7 and MDA-MB-231 cells were treated with ADR (0.5 µM) for 48 h, we conducted an analysis of DNA damage by comet assays and γ-H2AX foci formation. The images were captured using a fluorescence microscope at ×400 magnification. Comet tails represent fragmented DNA leaked from the nucleus. Tail intensity (% Tail DNA), defined as the percentage of Comet tails migrated from the head of the comet to the tail, was applied as a measure of DNA damage;^48^ 50 comet tails were analyzed for each sample. Quantification results are shown to the right. *p < 0.05 vs. MCF-7 group. For each sample, 50 nuclei were analyzed. **f** Western blot analysis of DNA repair-related proteins in BrCa cell with different p53 status. **g** MCF-7 and MDA-MB-231 cells were untreated and treated with ADR (0.5 µM) for 48 h. The cells were lysed for Western blotting and probed with the indicated antibodies. **h** Immunoblot analysis of MCF-7 cells transfected with an empty vector (control) or the p53R280K plasmid and then treated with ADR (0.5 µM) for 48 h. Data represent mean ± SD of three independent experiments. (*p < 0.05, **p < 0.01, ***p < 0.001)

To determine whether p53 status can affect the survival of BrCa cells upon ADR treatment, we first evaluated and compared the ADR sensitivity of a panel of BrCa cell lines with known p53 status. We found that cells expressing mutp53 (MDA-MB-231 and T-47D) were significantly more resistant to ADR than cells expressing wtp53 (MCF-10A, MCF-7, and ZR-75-1) (Fig. 1a, b). Strikingly, introducing the mutp53 R280K into wtp53 MCF-7 and ZR-75-1 cells reduced their ADR sensitivity (Fig. 1c). In contrast, the restoration of p53 expression sensitized the p53-mutated MDA-MB-231and T-47D cells to ADR (Fig. 1d).

Because DNA damage repair is one of the main mechanisms that affects the efficacy of chemotherapy, to define a mechanism of action for mutp53 in the response to ADR, we first compared the differences in DNA damage between p53 wt vs. p53 mut-type BrCa cells. We found that MCF-7 cells expressing wtp53 had significantly more residual DNA DSBs than MDA-MB-231 cells expressing mutp53 cells, as evidenced by the increased signal intensity of γ-H2AX staining and by the increase in comet tail moments (Fig. 1e). We next compared the expression of DNA repair pathway proteins in p53-mutated and p53-wt cell lines, and those with p53 mutations showed significantly increased levels of FANCF, REV1, RAD51, BRCA1, ATM, and ERCC1 compared to those of the p53 wild-type cell lines (Fig. 1f) and other genes including FANCM, ERCC4, RAD52, and REV3 have no significant differences (Supplementary Fig. S1). We subsequently focused on FANCF and REV1, the proteins with the most increased expression levels in the p53-mutant cell lines. Additionally, reduced levels of FANCF and REV1 were detected in ADR-treated MCF-7 cells but not in MDA-MB-231 cells (Fig. 1g), and the effect was largely impeded in p53-R280K-expressing cells (Fig. 1h). These data suggested that increased FANCF and REV1 levels may represent a molecular determinant for ADR resistance in p53-mutant cell lines.

### miR-30c inhibits FANCF and REV1 expression by targeting their 3′-UTRs

**Fig. 2:**
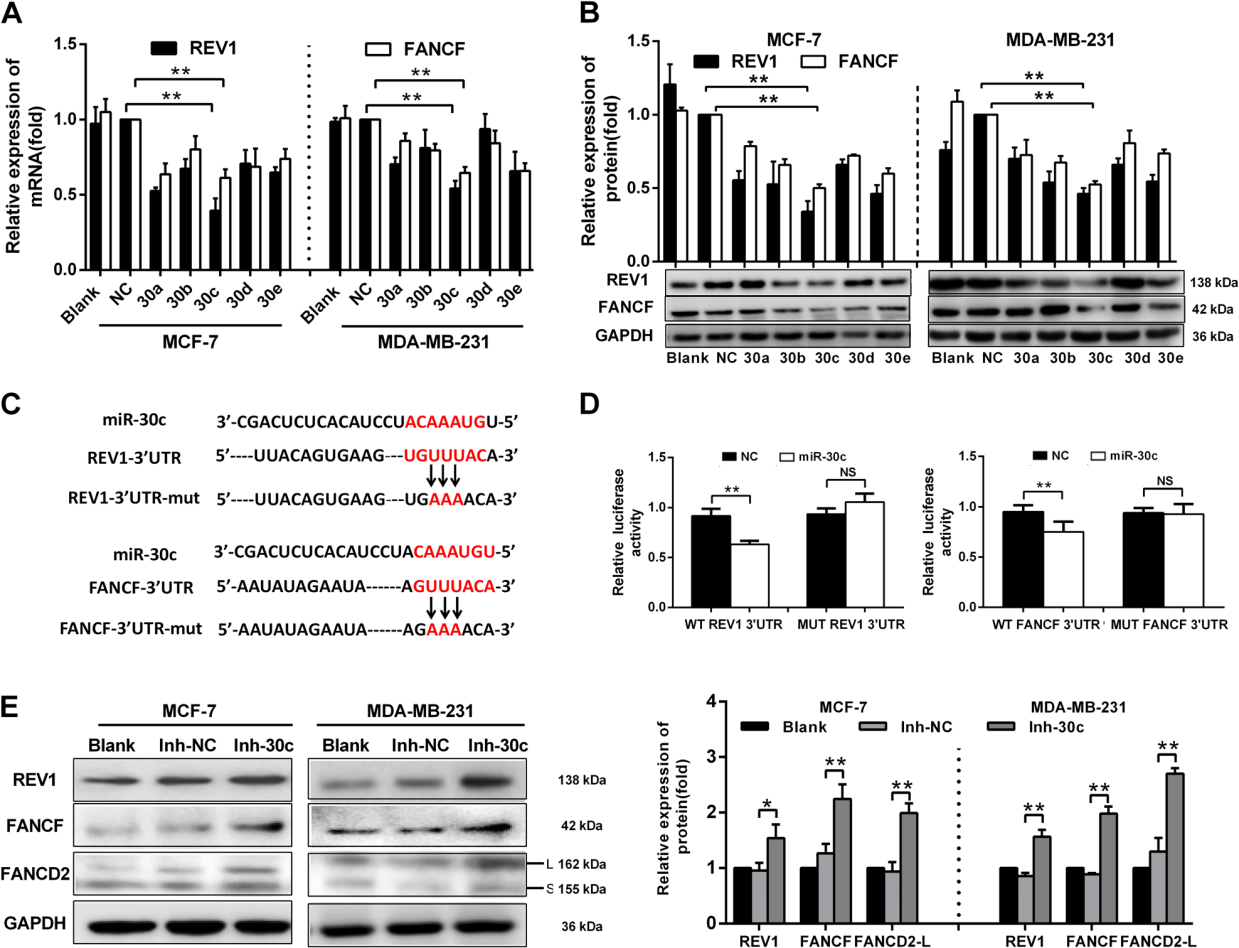
miR-30c inhibits FANCF and REV1 expression by targeting the 3′-UTRs of FANCF and REV1. **a** qRT-PCR and **b** Western blot analyses showing the mRNA and protein expression of REV1 and FANCF in untreated MCF-7 and MDA-MB-231 cells (blank), as well as cells transfected with miR-30a, miR-30b, miR-30c, miR-30d, miR-30e mimic and negative control (NC, scrambled control oligonucleotides). **p < 0.01 vs. NC group. **c** The REV1 and FANCF 3′UTR binding sites of miR-30c and the REV1 and FANCF 3′UTR mutation sites in the mutated luciferase plasmid. **d** Dual-luciferase activity in 293 T cells transfected with miR-30c mimic (20 nM), NC (20 nM) and a wild-type or mutated 3′UTR of REV1 or FANCF, **p < 0.01 vs. NC group. NS indicates no significant difference. NC: negative control, scrambled control oligonucleotides. **e** Western blot analysis showing the protein expression of REV1, FANCF, and FANCD2 in MCF-7 and MDA-MB-231 cells transfected with miR-30c inhibitors (20 nM) or the NC inhibitor (20 nM) and untreated MCF-7 and MDA-MB-231 cells (Blank). The graph showing the statistical results is located on the right. *p < 0.05, **p < 0.01 vs. Inh-NC group. Data represent the mean ± SD of three independent experiments

Since ADR resistance in p53-mutated BrCa cells is related to the high expression of FANCF and REV1, we used online databases (TargetScan, RNA2 and RNAhybrid) to screen for miRNAs that simultaneously target the 3′-UTRs of the FANCF and REV1 mRNAs. Only the miR-30 family (including miR-30a, miR-30b, miR-30c, miR-30d and miR-30e) contained putative binding sites for FANCF and REV1 (Supplementary Fig. S2ab). The minimum free energy between miR-30 and the putative binding sites in the 3′-UTRs of the REV1 and FANCF mRNAs suggested that miR-30 may target REV1 and FANCF (Supplementary Fig. S2c). We then detected the expression of REV1 and FANCF in MCF-7 and MDA-MB-231 cells transfected with miR-30 family miRNAs using qRT-PCR and Western blot analyses. The expression levels of REV1 and FANCF were significantly decreased in cells overexpressing miR-30 family miRNAs, and miR-30c produced the strongest effect (Fig. 2a, b). Furthermore, luciferase activity assays showed that miR-30c overexpression significantly reduced luciferase activity in cells transfected with the wild-type pGL3-REV1-3′UTR-Full and pGL3-FANCF-3′UTR-Full vectors. Mutations in the putative binding sites in the 3′-UTRs of the REV1 and FANCF mRNAs abolished the inhibitory effect of miR-30c on luciferase activity (Fig. 2c, d). The inhibition of endogenous miR-30c by miR-30c inhibitors increased the mRNA (Supplementary Fig. S3) and protein expression (Fig. 2e) of REV1 and FANCF in BrCa cells. miR-30c inhibitors also increased the expression of FANCD2-L (the monoubiquitylated isoform) and FANCD2-S (the primary unmodified translation product), which activates the FA/BRCA pathway^11^ (Fig. 2e). These results suggested that miR-30c directly targets REV1 and FANCF via the putative binding sites in the 3 ′-UTRs of the REV1 and FANCF mRNAs.

### miR-30c enhances chemosensitivity to ADR in BrCa cells in vitro and in vivo

**Fig. 3:**
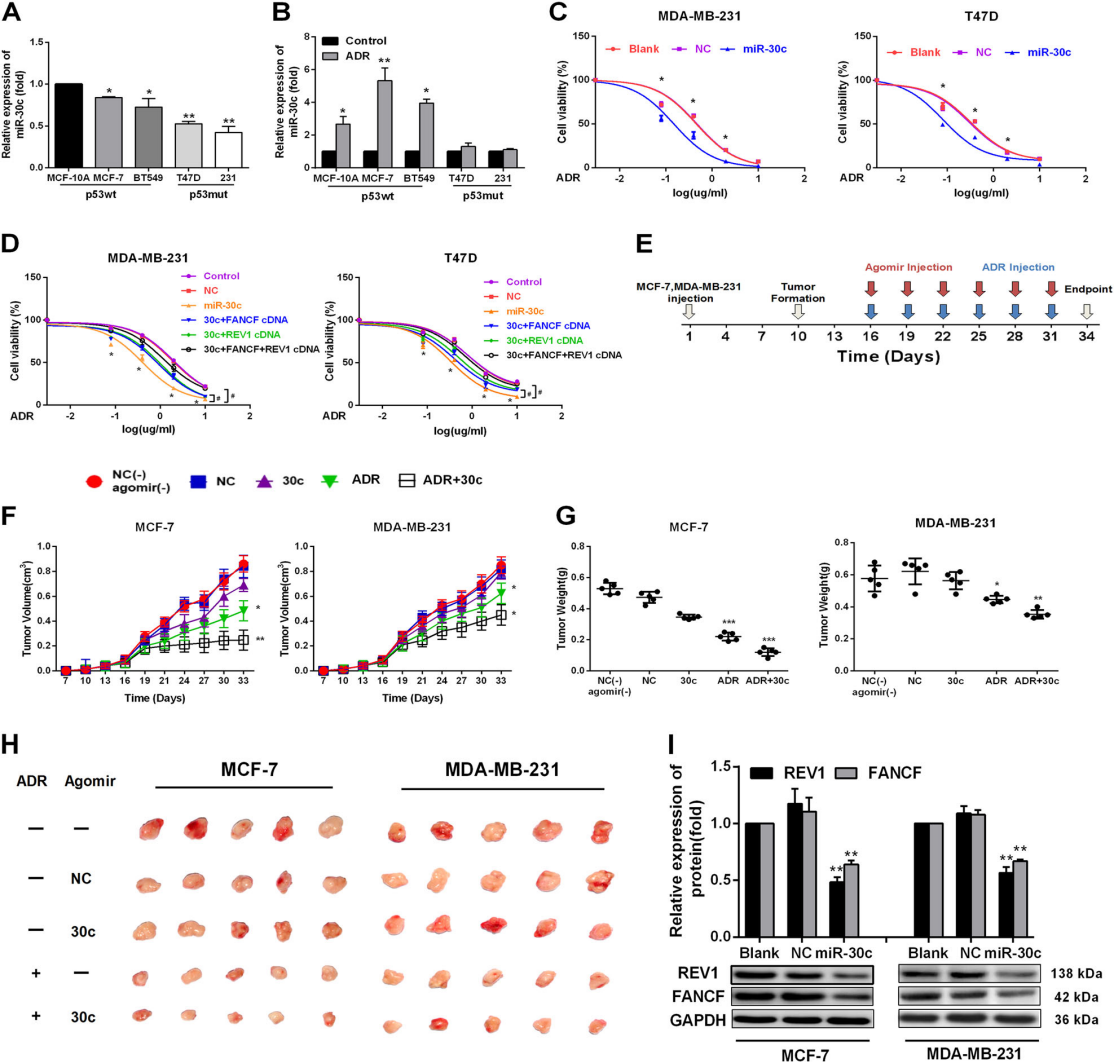
miR-30c potentiated the antitumor effect of ADR in vitro and in vivo. **a** Real-time PCR analysis of miR-30c basal expression in wtp53 (MCF-10A, MCF-7, and ZR-75-1) and mutp53 (MDA-MB-231, and T-47D) BrCa cells. *p < 0.05, wtp53 vs. mutp53 BrCa. **b** wtp53 and mutp53 BrCa cells were treated with ADR (0.5 µM) for 48 h. The expression of miR-30c was evaluated by qRT-PCR. *p < 0.05,**p < 0.01 vs. control group. NS indicates no significant difference. **c** MDA-MB-231 and T47D cells were transiently transfected with the NC or miR-30c mimic and were treated with various ADR concentrations. The relative viability of the MDA-MB-231 and T47D cells was determined via CCK-8 assays (*n* = 3). *p < 0.05 vs. NC group. **d** The relative viability of the MDA-MB-231and T47D cells was detected by CCK-8 assays 48 h after transfection with NC (20 nM), miR-30c mimic (20 nM), FANCF (4 µg), REV1 cDNA(4 µg) or FANCF and REV1 cDNA. FANCF and REV1 constructs are plasmids without 3′ UTR region. ^**#**^*p* < 0.05 vs. miR-30c group, *p < 0.05 vs. NC group. **e** MCF-7 and MDA-MB-231 cells were injected subcutaneously into nude mice. A cholesterol-conjugated miR-30c agomir or negative control (NC) agomir was intratumorally injected 7 days after tumor formation, and agomir and ADR were intratumorally injected every two days (*n* = 5). **f** Xenograft tumor growth was monitored. ^**#**^*p* < 0.05, ^**##**^*p* < 0.01 vs. ADR group; *p < 0.05, **p < 0.01 vs. NC(-) agomir group. **g** Tumor weight at sacrifice. *p < 0.05, ***p < 0.001 vs. NC(−) agomir group; ^**#**^*p* < 0.05, ^**###**^*p* < 0.001 vs. ADR group. Data represent the mean ± SD (*n* = 5, each group). **h** Photographs of the removed tumors at day 34 after the implantation. **i** Western blot analysis for FANCF and REV1 in the MCF-7 and MDA-MB-231 xenografts after treatment with the cholesterol-conjugated miR-30c agomir or the NC agomir. **p < 0.01 vs. NC group

As miR-30c downregulates FANCF and REV1, we wondered if this miRNA could sensitize cells to ADR. We found that endogenous miR-30c was highly expressed in ADR-sensitive p53wt BrCa cells (Fig. 3a). Moreover, ADR significantly increased the levels of miR-30c in p53 wt cells but not in p53-mutated cells (Fig. 3b), suggesting a positive correlation between the miR-30c expression level and sensitivity to ADR in BrCa cells. Furthermore, overexpression of miR-30c significantly increased chemosensitivity to ADR in both MDA-MB-231 and T-47D cells (Fig. 3c). Conversely, miR-30c inhibition significantly decreased chemosensitivity to ADR in both MCF-7 and ZR-75-1 cells (Supplementary Fig. S4). To determine the functional significance of FANCF and REV1 suppression in miR-30c-mediated chemotherapeutic resistance, we transfected MDA-MB-231 and T-47D cells with miR-30c alone or in combination with a FANCF and REV1 construct without 3′ UTR region. The reduced cell survival caused by miR-30c overexpression in ADR-treated MDA-MB-231 and T-47D cells was significantly attenuated by the co-transfection of FANCF and REV1cDNA without 3′ UTR region (Fig. 3d). To further validate the importance of FANCF/REV1 in ADR resistance mutp53 cells, we performed the experiment of the effect of the FANCF/REV1 on ADR resistance without overexpressing miR30c in MDA-MB-231 cell and MCF-7 cell. Compared with the control group, ADR sensitivity increased significantly after silencing FANCF and REV1 in MDA-MB-231 cell (shown in Supplementary Fig. S5) and decreased significantly after FANCF and REV1 overexpression in MCF-7 cell (Supplementary Fig. S6). And the increased cell survival caused by p53R280K overexpression in ADR-treated MCF-7 cells was significantly attenuated by the co-transfection of FANCF and REV1 siRNA(Supplementary Fig. S7). These results suggested that FANCF + REV1 could be used to target ADR-resistant mutp53 cells and miR-30c enhances the drug sensitivity of BrCa cells to ADR by regulating the expression of REV1 and FANCF.

To validate the potential pathophysiological significance of miR-30c induction in the therapeutic response of BrCa to ADR, we next examined the effect of miR-30c on FANCF and REV1 expression and chemosensitivity in nude mice bearing human MCF-7 and MDA-MB-231 xenografts, using cholesterol-conjugated miR-30c mimics (miR-30c agomir) or ADR (Fig. 3e). ADR significantly delayed the growth of MCF-7 and MDA-MB-231 xenograft tumors (Fig. 3f–h). Moreover, miR-30c treatment in combination with ADR significantly reduced the tumor volume and tumor weight at sacrifice compared with those from mice treated with ADR alone, suggesting that miR-30c had a chemosensitizing effect (Fig. 3f–h). Furthermore, the expression of FANCF and REV1 was significantly reduced in miR-30c-overexpressing tumors (Fig. 3i and Supplementary Fig. S8). Taken together, these findings demonstrated that miR-30c enhances the therapeutic activity of ADR in drug-resistant and drug-sensitive cells by targeting FANCF and REV1.

### miR-30c disturbs the DNA damage response in breast cancer cells

**Fig. 4:**
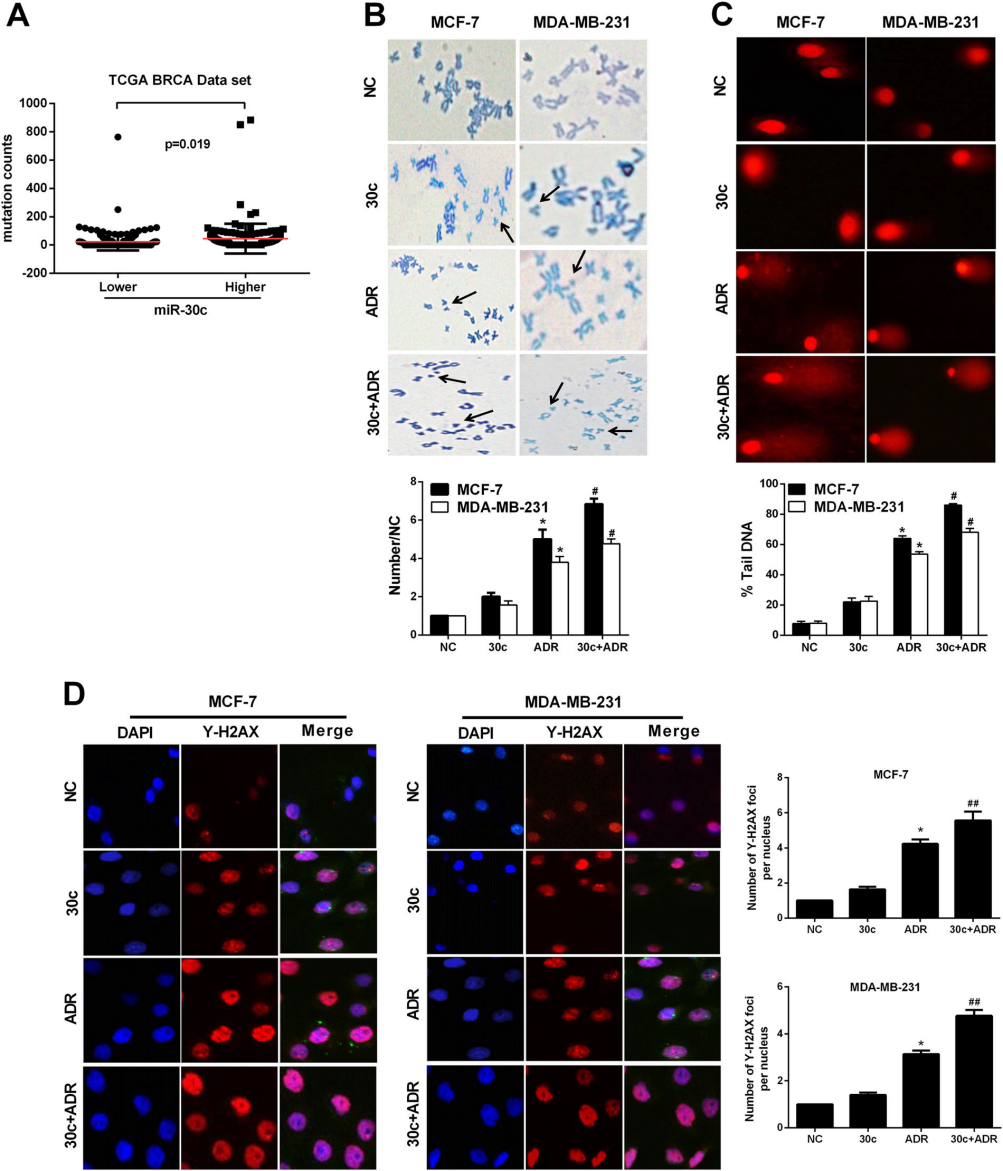
miR-30c enhanced DNA damage and genome instability following ADR treatment. **a** Genomic instability (mutation counts) was detected in TCGA BrCa patients (*p* < 0.05, *n* = 352). **b, c** MCF-7 and MDA-MB-231 cells were transfected with a negative control (NC) mimic (20 nM), miR-30c mimic (20 nM), ADR (0.5 µM) or miR-30c + ADR for 48 h. Chromosomes were then subjected to cytogenetic analysis. A total of 50 cells from each sample was examined and scored. Arrowheads indicate chromosome breaks. Magnification, ×400. For each sample, 50 comet tails were analyzed. *p < 0.05 vs. NC group, ^#^*p* < 0.05 vs. ADR group. **d** Representative immunofluorescent images of γ-H2AX staining in MCF-7 and MDA-MB-231 cells treated with a negative control (NC) mimic (20 nM), miR-30c (20 nM), ADR(0.5 µM), or miR-30c + ADR for 48 h. Nuclei were counterstained with DAPI. For each group, 50 nuclei were analyzed. Magnification, ×200. Quantification results are shown on the right. *p < 0.05 vs. NC group, ^##^*p* < 0.01 vs. ADR group. Data represent the mean ± SD (*n* = 3, each group)

FANCF and REV1 are two key factors in DNA damage repair pathways^12,13^. In the Cancer Genome Atlas (TCGA) dataset, BrCa patients with higher levels of miR-30c have more DNA mutations than cancer patients with lower miR-30c expression (Fig. 4a), indicating that miR-30c increases genomic instability. Therefore, we examined the effect of miR-30c on DNA damage repair through chromosome fracture analysis and alkaline COMET assays. miR-30c overexpression increased the number of chromosomal breakages as well as comet tail length in the presence and absence of ADR in MCF-7 and MDA-MB-231 cells (Fig. 4b, c).

DSBs can induce the phosphorylation of H2AX, forming γ-H2AX, γ-H2AX foci represent the number of DSBs^14,15^. The number of γ-H2AX foci induced by ADR treatment were significantly increased in miR-30c-overexpressing cells (Fig. 4d), suggesting that miR-30c delays DNA damage repair.

### p53 regulates REV1 and FANCF through the activation of miR-30c transcription by binding to the promoter region of miR-30c

**Fig. 5:**
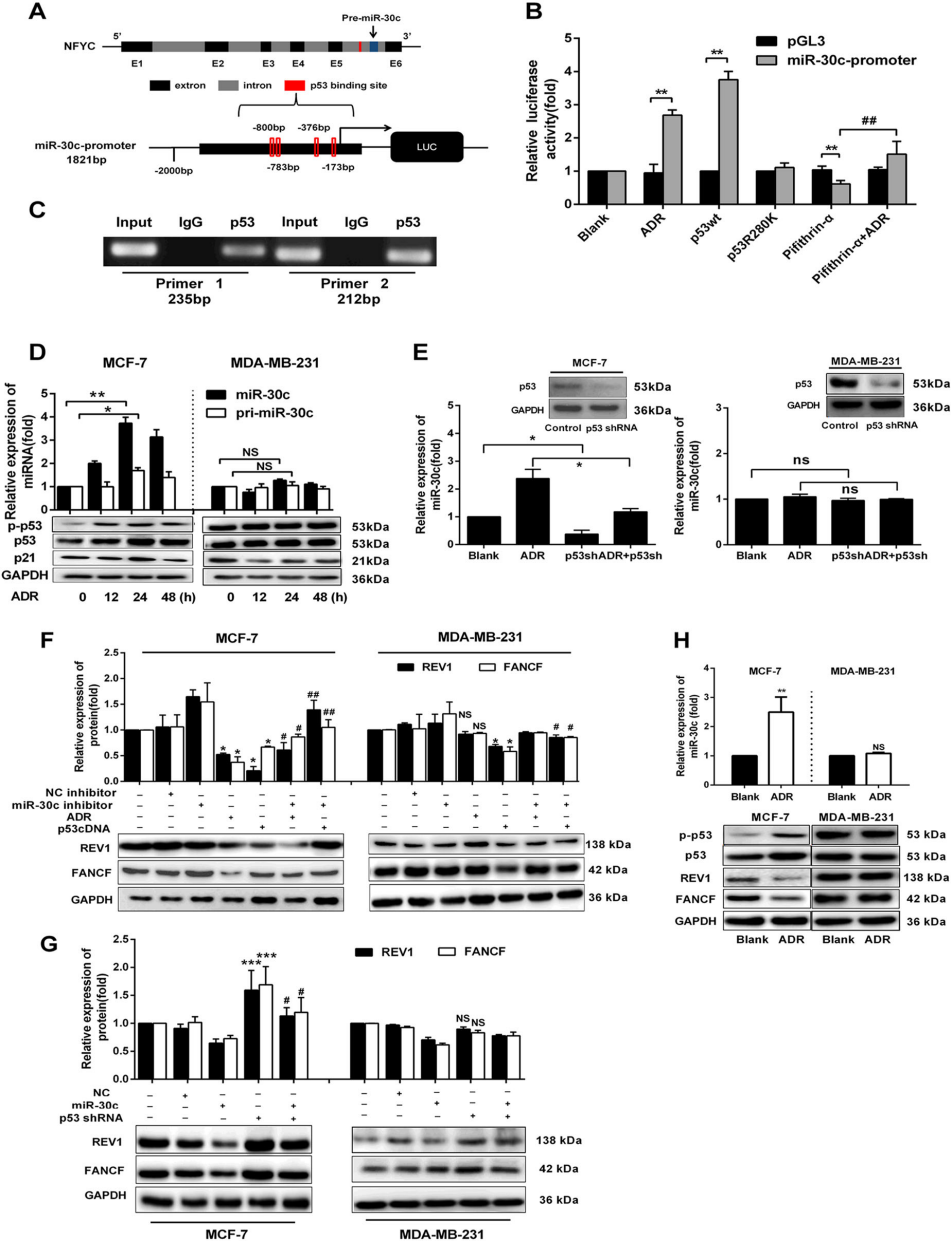
p53 regulates the expression of FANCF and REV1 via miR-30c in BrCa. **a** Schematic representation of *NFYC* (miR-30c host gene) and putative p53 binding sites in intron 5 of *NFYC*. **b** HEK-293T cells were treated with pcDNA3.1-p53 (wtp53), pcDNA3.1-p53R280K (mutp53), ADR (0.5 µM), pifithrin-α (10 µM), pifithrin-α + ADR or the miR-30c promoter constructs (in the pGL3 vector). Relative luciferase activity was assayed. **p < 0.01 vs. PGL3 vector, ^##^*p* < 0.01 vs. pifithrin-α group. **c** ChIP assay showing endogenous p53 bound to the miR-30c promoter in the p53 binding site region in MCF-7 cells. **d** MCF-7 and MDA-MB-231 cells were treated with ADR (0.5 µM) for 12, 24, and 48 h. The expression of mature miR-30c and pri-miR-30c was evaluated by qRT-PCR. The protein expression of p-p53, p53, and p21 was examined by Western blotting. *p < 0.05, **p < 0.01 vs. 0 h group. **e** MCF-7 and MDA-MB-231 cells were treated with control, p53shRNA, ADR, or p53shRNA + ADR for 48 h. qRT-PCR showed that the miR-30c expression changes. The protein expression of p53 was examined by Western blotting. **f** MCF-7 and MDA-MB-231 cells were transfected with NC inhibitor (20 nM) or miR-30c inhibitor (20 nM) and were then treated with ADR and p53cDNA. The expression of FANCF and REV1 was analyzed by Western blotting. MCF-7 cells: *p < 0.05, **p < 0.01 vs. Blank group; ^#^*p* < 0.05 vs. ADR group, ^##^*p* < 0.01, vs. p53cDNA group. MDA-MD-231 cells: *p < 0.05 vs. Blank group; ^#^*p* < 0.05 vs. p53cDNA group. **g** FANCF and REV1 expression in MCF-7 and MDA-MB-231 cells transfected with p53 shRNA, miR-30c mimic (miR-30c) or the negative control (NC). The expression of FANCF and REV1 was analyzed by Western blotting. **p < 0.01, ***p < 0.001 vs. Blank group; ^#^*p* < 0.05 vs. Blank group. **h** qRT-PCR analysis of miR-30c expression in ADR-treated MCF-7 and MDA-MB-231 xenografts. Western blot analysis of p-p53, p53, FANCF, and REV1 expression in ADR-treated MCF-7 and MDA-MB-231 xenografts. **p < 0.01, ***p < 0.001 vs. Blank group. NS indicates no significant difference. Data represent the mean ± SD (*n* = 3, each group)

We further searched online databases (http://www.biobase-international.com/ Gene-regulation) to identify transcription factors that regulate miR-30c transcription. We found p53 binds to the promoter region of miR-30c, with its binding site is located within intron 5 of *NFYC* (the miR-30c host gene) on GRCh38.p2 of chromosome 1 (Fig. 5a). Luciferase activity assays showed that the overexpression of wtp53, but not mutated p53, significantly increased the luciferase activity. Moreover, in comparison with the transfection of wtp53 (p53cDNA), luciferase activity induction was diminished in cells transfected with mutp53 (p53R280K) (Fig. 5b). Furthermore, ADR, a genotoxic agent that activates p53, increased luciferase activity, and pifithrin-α, a specific p53 inhibitor, inhibited the p53-induced increased luciferase activity. However, pifithrin-α and ADR treatment significantly increased the luciferase activity after pifithrin-α treatment alone in HEK-293T and MCF-7 cells (Fig. 5b and Supplementary Fig. S9). ChIP assays confirmed that p53 directly binds to the identified binding site of the miR-30c promoter in vivo (Fig. 5c). Further, we found that ADR, which significantly induced the expression of p53, significantly increased the levels of miR-30c and pri-miR-30c in MCF-7 but not in MDA-MB-231 cell (Fig. 5d). Similarly, the specific p53 agonist nutlin-3 also increased the expression of miR-30c and pri-miR-30c in MCF-7 but not in MDA-MB-231 cell (Supplementary Fig. S10a). The overexpression of wtp53 increased miR-30c expression in both MCF-7 and MDA-MB-231 cells (Supplementary Fig. S10b). Furthermore, p53 shRNA significantly reduced the expression of miR-30c in the presence or absence of ADR. (Fig. 5e).

Our results indicated that miR-30c is regulated at the transcriptional level. As positive and negative controls, we tested two p53 targets with or without treating the cells with the translation inhibitor cycloheximide. The direct transcriptional target gene p21CIP1/WAF1 was upregulated after the induction of wtp53 irrespective of functional translation(Supplementary Fig. S11a). As an example of a gene indirectly controlled by p53, cyclin B2 mRNA was downregulated, but it did not change significantly when translation was inhibited (Supplementary Fig. S11b). Interestingly, the levels of miR-30c were induced after p53 expression independent of functional translation (Supplementary Fig. S11c), which supports the notion that the miR-30c gene locus is a direct target of p53.

We further examined whether p53 regulates REV1 and FANCF via miR-30c in BrCa cells. We found that wtp53 overexpression significantly suppressed the expression of FANCF and REV1 in MCF-7 and MDA-MB-231 cells. p53 activation induced by ADR suppressed the expression of FANCF and REV1 in MCF-7 but not in MDA-MB-231 cell (Fig. 5f). miR-30c inhibitors inhibited the effects of wtp53 overexpression and ADR on the expression of FANCF and REV1 (Fig. 5f). In addition, p53 knockdown by shRNA significantly increased the expression of REV1 and FANCF in MCF-7 cell, and this effect was blocked by miR-30c mimics (Fig. 5g). In line with these in vitro results, we also observed that ADR treatment significantly increased the miR-30c levels and decreased the expression of FANCF and REV1 in MCF-7 but not in MDA-MB-231 xenograft tumors (Fig. 5h, Supplementary Fig. S12). Therefore, our data suggest that REV1 and FANCF are regulated by wtp53 through miR-30c.

### Reduced miR-30c expression is highly correlated with human BrCa p53 mutational status and is associated with poor survival

**Fig. 6:**
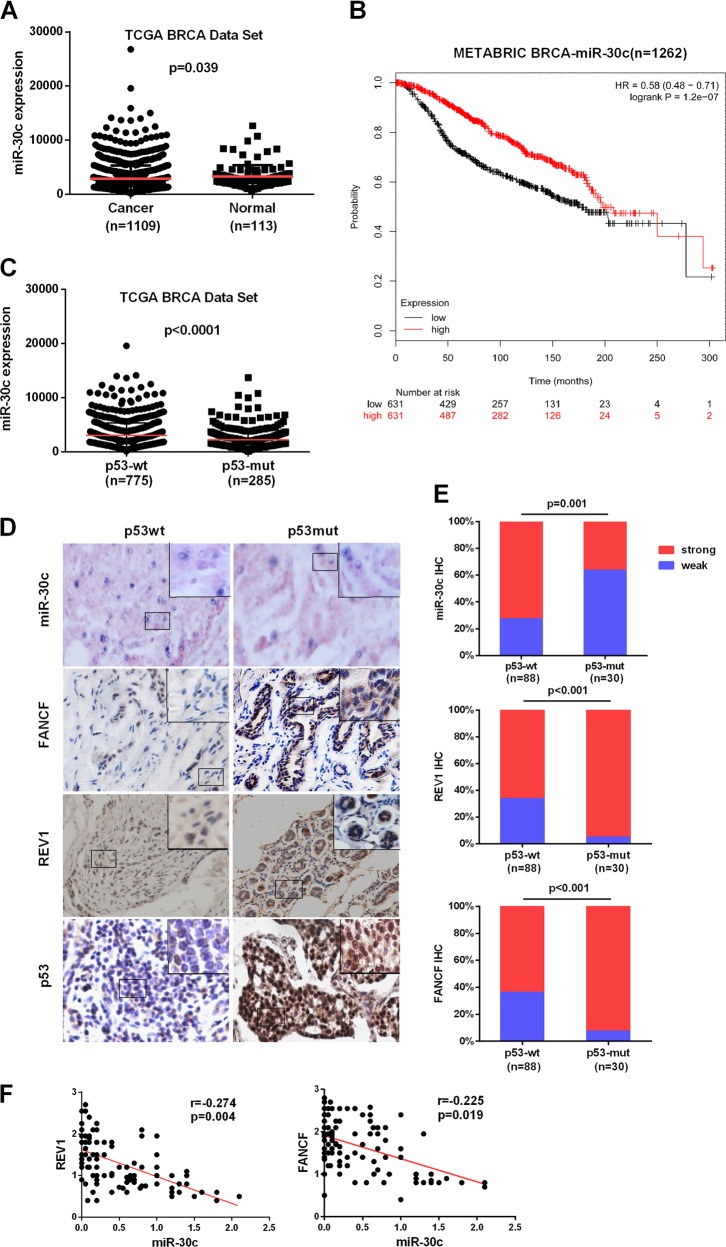
Expression of miR-30c, FANCF and REV1 proteins is elevated in mutp53 specimens from patients with BrCa. **a** miR-30c expression in normal breast tissues (*n* = 113) and BrCa tissues (*n* = 1109) in The Cancer Genome Atlas (TCGA) dataset. The p value was calculated via a nonparametric Mann-Whitney test. **b** Kaplan–Meier overall survival curves according to miR-30c expression in METABRIC BrCa patient cohorts. **c** A whisker plot showing miR-30c expression in patients with mutp53 tumors (*n* = 285) and wtp53 tumors (n = 775) using TCGA database. **d** Representative images of miR-30c *in situ* hybridization and FANCF and REV1 IHC for 118 cases of BrCa expressing wtp53 and mutp53. Magnification, ×200. Small frames indicate the magnified regions. **e** Quantitative data for miR-30c, FANCF and REV1 protein staining in **d**. Statistical significance was determined by Wilcoxon rank-sum tests. **f** Correlation analysis of miR-30c and REV1 or FANCF protein expression in BrCa patients

The above results predict that tumors with mutated p53 should have low expression of miR-30c and high expression of FANCF and REV1. To investigate any potential association among miR-30c expression, p53 mutation status, and clinical BrCa data, we examined a BrCa RNAseq dataset from TCGA. We observed a significant difference in miR-30c expression between tumors (*n* = 1109) and normal tissues (*n* = 113) (Fig. 6a). Kaplan–Meier survival analysis showed a highly significant difference in overall survival between the high expression (*n* = 631) and low expression (*n* = 631) groups. Elevated miR-30c expression significantly correlated with a longer survival time in BrCa patients (*p* < 0.001, Fig. 6b). We then compared miR-30c expression in mutp53 vs wtp53 tumors in patients with BrCa using the TCGA BrCa database. Tumors with mutated p53 had lower miR-30c expression than those with wtp53 (*p* < 0.001, Fig. 6c). To further examine the effect of p53 mutations on miR-30c levels and FANCF and REV1 protein levels in specimens from individuals with BrCa, we analyzed miR-30c, FANCF and REV1 in our cohort for which 118 primary breast tumor samples were available. The presence of potential p53 gain-of-function mutations was inferred by strong p53 staining via IHC (Fig. 6d). Consistent with the TCGA miRNA-seq dataset analysis, miR-30c expression was markedly higher in tumor tissues with wtp53 (Fig. 6e). IHC analysis showed that p53-mutated tumors exhibited high staining for FANCF and REV1. In contrast, tumors with wtp53 exhibited low staining for the FANCF and REV1 proteins (Fig. 6e). Spearman correlation analysis showed that FANCF and REV1 protein expression was negatively correlated with miR-30c expression in BrCa tissues (Fig. 6f). These observations support the notion that p53 activates miR-30c to inhibit REV1 and FANCF expression. miR-30c has prognostic value and may play an important role in cancer progression.

## Discussion

Chemotherapeutic resistance, particularly to ADR, represents a major impediment to successfully treating BrCa. Currently, no predictive biomarkers for ADR resistance have been identified for general clinical use. As the most frequently mutated gene in human tumors^3^, p53 mutations contribute to resistance to a variety of standard chemotherapies^16–18^. While p53 mutational status has been linked to a lack of sensitivity to anthracyclines^19,20^, its correlation with resistance to ADR-based chemotherapeutics has not always been straightforward^21–24^, with studies showing variable responses. It is possibly due to molecular changes, especially to molecules up- or downstream of mutp53 networks. Furthermore, although drugs targeting mutp53 have been developed^17^, their efficacy in the treatment of human cancer is unclear. Therefore, exploring the molecules involved in mutp53 networks may facilitate the prediction of chemotherapy response as well as the development of individualized chemotherapy for tumors with p53 mutations in the future. In this regard, our study highlights a mechanism of intrinsic ADR resistance in p53-mutated BrCa involving miR-30c/FANCF/REV1-mediated DNA damage response.

To date, different molecular mechanisms of action underlying mutant p53 gain-of-function have been described^25^. DNA repair mechanisms are considered a vital target for improving cancer therapy and reducing resistance to many DNA-damaging agents currently in use as standard-of-care treatments. In our study, we focused on the role of DNA repair in ADR resistance in p53-mutated BrCa. We found that FANCF and REV1, which are two important DNA repair genes, are increased the most in p53-mutatated BrCa cell lines compared to wtp53 cell lines. The FA/BRCA pathway is involved in the maintenance of cell growth, proliferation, and apoptosis^26,27^. FANCF is critically involved in regulating the function of the FA/BRCA pathway by maintaining the stability of the FA core complex as well as the ubiquitin activation (monoubiquitination) of the FANCD2 protein^28^. Our previous studies found that the inhibition of FANCF blocked the functions of FA/BRCA pathway and enhanced antitumor drug sensitivity in cancer cells^29–32^. REV1-mediated TLS may play a critical role in the development of acquired chemoresistance^33^ and improving chemotherapeutics^34^. Therefore, the simultaneous inhibition of FANCF and REV1 is a theoretically valid strategy for sensitizing tumor cells to DNA-damaging agents and preventing the development of chemoresistance; however, one concern is that this combination may also lead to toxicity in some normal tissues.

miR-30c as a tumor suppressor, the current studies demonstrate that miR-30c plays a role in chemoresistance by regulating the anti-apoptotic gene YWHAZ^35^ and epithelial–mesenchymal transition (EMT) related genes TWF1^36^. In addition to its role in regulating chemoresistance, miR-30c also regulates embryo development through downregulation of several tested DNA damage response (DDR) genes^37^. Here, we revealed a new role for miR-30c as a tumor suppressor:^38–40^ miR-30c may provide a way for cells to shut down DNA repair machinery after extensive damage. Moreover, the increased miR-30c level without ADR did not affect cancer cell growth (Supplementary Fig. S13), which is consistent with the results reported by Bockhorn J^36^. Thus, miR-30c, which downregulates both FANCF and REV1, is a potentially powerful therapeutic agent for improving the efficacy of ADR. However, miR-30c intervention alone is insufficient to reverse ADR resistance and other signaling pathways may also play a significant role in ADR resistance in p53-mutant cells. Our findings provided a clue to the ADR resistance of p53 mutations and outline a novel pathway for interrupting DNA damage repair to enhance the anti-cancer function of ADR.

More importantly, our work reveals that miR-30c is a target of p53 and that p53 regulates miR-30c transcriptional activation. A previously published analysis revealed that a cohort of miRNAs exhibit p53-dependent upregulation following DNA damage and defined miR-30c as one of the potential targets of p53^41^. Since the p53 conserved sequence matches to the miR-30c promoter region, this binding also presents in other tumors (Supplementary Fig. S14). Most p53 mutations found in cancers are located in a domain that is required for both its miRNA processing function and transcriptional activity^42,43^. As expected, mutation of p53 abolished this response. Thus, our data suggest that p53 mutation-conferred resistance to ADR might be related to the decreased miR-30c levels and increased FANCF and REV1 protein levels in BrCa cells.

It is well known that oncogenic activities acquired by mutant p53 include increased proliferation and acquisition of resistance to specific therapies^44^. REV1/FANCF as a DNA damage response gene has previously been shown to be induced by DNA damage. Unexpected, we found that Rev1 and FANCF are decreased upon ADR treatment in MCF-7 cell.These findings seemed to suggest that REV1 and FANCF failed to really repair the ADR-induced DNA damage in our study. We detected the expression of REV1 and FANCF at ADR different concentrations and different treatment times in MCF-7 cell. As shown in Supplementary Fig. S15& S16, when the ADR concentration is small or the treatment time is short, the DNA damage repair pathway is activated, the cells intend to repair the DNA and survive. When the concentration of ADR is large or the treatment time is longer, the expression of REV1 and FANCF is decreased in breast cancer cells. Therefore, this may be the reason why our results are inconsistent with others.

In conclusion, our work suggests a role for miR-30c in the regulation of DNA repair through the modulation of FANCF and REV1 expression. Our findings highlight the functions and roles of miRNAs in BrCa and suggest that resistance to ADR in p53-mutant BrCa may be related to miR-30c/FANCF/REV1-mediated DNA damage response.

## Materials and methods

### Cell culture

The BrCa cell lines MCF-7, ZR-75-1, T-47D, MDA-MB-231 (ATCC, USA) and MCF-10A (ATCC, USA) were cultured under standard conditions, as recommended by their manufacturers. pMKO.1 puro p53 shRNA (Plasmid #10671) and pCMV-Neo-Bam p53wt (Plasmid #16434) were obtained from Addgene (http://www.addgene.org). p53R280K cDNA, REV1 cDNA and FANCF cDNA plasmids without 3′ UTR region were obtained from GeneChem (www.genechem.com.cn).

### Tissue samples

Formalin-fixed and paraffin-embedded BrCa samples were obtained from the Department of Surgical Oncology at the First Affiliated Hospital of China Medical University. None of the patients had received radiotherapy or chemotherapy before surgery. The use of human tissues was approved by the ethics committee of China Medical University.

### miRNA and RNA interference

miR-30c mimic (miR-30c), miR-30c negative control (miR-NC), miR-30c inhibitor (Inh-30c), miR-30c inhibitor negative control (Inh-NC) and negative control siRNAs (siRNA duplexes with non-specific sequences) were synthesized and purified by Riobio (Riobio, Co., Guangzhou, China). Cells were transfected with miR-30c (20 nM), Inh-30c (20 nM), REV1 siRNA (20 nM) or FANCF siRNA (20 nM) using Lipofectamine 2000. The expression levels of miR-30c and other mRNAs were quantified 48 h after transfection. REV1 siRNA and FANCF siRNA were obtained from Riobio.

### Cell proliferation assay

Cells were transfected with p53R280K(4ug), p53cDNA(4ug), miR-30c mimics (20 nM), miR-30cinhibitor(20 nM), FANCFcDNA/REV1cDNA(4ug), or FANCFsiRNA / REV1siRNA (20 nM) for 24 h. Cells were then seeded onto 96-well plates at a density of 4 × 10^3^ cells/well. After culture for 24 h, cells were treated with serial dilutions of ADR, followed by treatment with CCK-8 (Dojindo Molecular Technologies Inc., Japan) for 1 h. The absorbance at 450 nm was measured using a multi-mode reader (LD942, Beijing, China).

### Immunoblot and reporter assays

The immunoblot and reporter assays were performed as described previously^45^. The antibodies used are described in Supplementary Table S2. Bands were visualized with a chemiluminescent detecting system (Amersham, Freiburg, Germany). The intensity of the western blot bands was analyzed by ImageJ software (http://rsb.info.nih.gov/ij).

The human REV1-3′-UTR (766 bp) and FANCF-3′-UTR (2362 bp) was amplified by PCR and cloned into the XbaI site of the pGL3 vector (Promega). Mutations in the miRNA-binding site were generated using PCR-based mutagenesis (Takara, Dalian, China). To generate miR-30c promoter plasmids, the miR-30c promoter regions (−1832 to + 150 bp) were amplified by PCR and cloned to the pGL3-Basic vector (Promega, WI, USA), respectively. The primers used are listed in Supplementary Table S1.

To study the effect of miR-30c on FANCF and REV1 expression via targeting the 3′-UTRs of FANCF and REV1, Luciferase reporter vectors (control) or vectors containing wild-type (pGL3-REV1-3′UTR-Full, pGL3-FANCF-3′UTR-Full) or mutated 3′-UTRs (pGL3-REV1-3′UTR-Mut, pGL3-FANCF-3′UTR-Mut) of REV1 and FANCF mRNAs were cotransfected into 293 T cells with miR-30c, using Lipofectamine 2000. To study the direct binding of p53 to the promoter region of miR-30c, the miR-30c-promoter reporter constructs were cotransfected with p53cDNAwt, or p53cDNA-R280K into HEK-293T/MCF-7/HCT-116/OVCAR3 cells using Lipofectamine 2000. After transfection for 48 h, luciferase activity was detected using the Dual Luciferase Reporter Gene Assay kit (Promega). Relative luciferase activity normalized to the control.

### Quantitative real-time PCR

Total RNA extraction from cells and quantitative real-time PCR were performed as described previously^45^. Total RNA was isolated from tumor cells using TRIZOl reagents (Invitrogen, CA, USA) according to the manufacture’s protocol. qRT-PCR were performed using SYBR Premix ExTaq™ II kit (Takara). The primers are listed in Supplementary Table S2. Relative expression of miR-30c was normalized to U6 expression and REV1 and FANCF was to GAPDH. The fold change for each RNA relative to the control was calculated using the 2^−△△Ct^ method.

### Immunohistochemistry

For immunohistochemistry, slides were deparaffinized and rehydrated. Antigen retrieval was performed by a pressure cooker for 10 min in 0.01 M citrate buffer (pH 6.0), followed by treatment with 3% hydrogen peroxide for 5 min. Next, slides were blocked in sheep serum for 30 min, and then incubated with antibodies specific for REV1 (Rabbit Polyclonal 1:50 dilution, novus) and FANCF (Rabbit Polyclonal 1:200 dilution, Bioss) overnight at 4 °C. Immunostaining was performed using DAB according to the manufacturer’s instructions. The slides were mounted and the images were captured and analyzed by a fluorescence microscope.

### MiR-30c in situ hybridization

Paraffin sections were mounted on Super frost + glass slides and deparaffinized. The slides were then treated with proteinase-K 10 μg/ml at 37 °C for 10 min, pre-hybridizd in Exiqon hybridization buffer (Exiqon, Vedbæk, Denmark) at 37 °C for 2 h, hybridized with 50 nM miRNA-30c probe and washed stringently with 5 × SSC, 1 × SSC and 0.2 × SSC buffers at 37 °C for 30 min. DIG blocking reagent (Roche, Mannheim, Germany) was added for 1 h at 37 °C in maleic acid buffer with 2% sheep serum. After which, alkaline phosphatase-conjugated anti-digoxigenin was added at 4 °C for 12 h (1:500 in Roche blocking reagent). 4-nitroblue tetrazolium (NBT) and 5-brom-4-chloro-3′-Indolylphosphate (BCIP) substrate (Roche) were used for enzymatic development to form dark-blue NBT-formazan precipitates at 37 °C for 60 min. The sections were lightly counterstained with nuclear fast red (Vector Laboratories, Burlingname, CA) at 25 °C for 1 min and mounted. The expression of miR-30c was detected, using digoxin-labelled locked nucleic acid-modified RNA probes against the full length mature miR-30c sequence. MiR-30c probe sequences are listed in Table S1.

### Chromosomal breakage analysis

Cells were transfected with the miR-30c mimic (20 nM) and treated with ADR (0.5 µM) for 48 h. Subsequently, colcemid was added at a final concentration of 100 ng/ml and incubated for 90 min, after which the cells were treated with a hypotonic solution (0.075 M KCL) for 30 min. The cells were fixed with 3:1 methanol/acetic acid and stained with Giemsa stain, and 50 metaphase spreads per group were used to evaluate chromosomal breakage.

### Single-cell alkaline electrophoretic (comet) assays

DNA damage was assessed via a single-cell gel electrophoresis assay under alkaline conditions using a Comet Assay kit according to the manufacturer’s protocol (Trevigen, Gaithersburg, MD, USA, 4250-050-K). Briefly, cells were harvested 48 h after miR-30c mimic transfection and ADR treatment. Each cell sample was mixed with low melting point agarose and plated on a CometSlide. Subsequently, the cells were lysed overnight at 4 °C, subjected to electrophoresis at 25 V and 300 mA for 20 min under alkaline conditions, and stained with EB staining solution. Fifty cells were randomly chosen to score each sample. The percentage of Tail DNA was determined using Comet IV analysis software (Perceptive Instruments Ltd., Bury St. Edmunds, UK).

### Immunofluorescence staining

Cells treated with ADR were fixed for immunofluorescence staining as previously described^46^. Mouse anti-human γ-H2AX conjugated to Alexa Fluor 555 goat anti-mouse IgG was used (Supplementary Table S2). 40,6-diamidino-2-phenylindole (DAPI) (Sigma) was used to stain the nuclei. Isotype controls were used for all assays. Immunostaining was analyzed using a fluorescence microscope (Nikon, Japan). Approximately 50 cells were scored for the presence of foci per experimental group.

### Chromatin immunoprecipitation (ChIP) assay

ChIP assays were performed using a commercially available kit (Millipore, Merck KGaA, Darmstadt, Germany), according to the manufacturer’s instructions. Briefly, cells (2 × 10^6^) were crosslinked in 1% formaldehyde for 10 min at room temperature, with the crosslinking stopped by the addition of 0.125 M glycine. A primary antibody against p53 (Cell Signaling Technology, Danvers, MA. USA) was used to capture the chromatin. After overnight capture at 4 °C, the chromatin was collected, purified, and de-crosslinked at 65 °C. The precipitated DNA fragments were quantified by PCR analysis, using the primers listed in Supplementary Table S2.

### Animal study

All animal work was performed in accordance with a protocol approved by the Animal Center and Animal Ethics Committee of China Medical University^47^. To establish breast tumor xenograft model, MCF-7 and MDA-MB-231cells (5 × 10^6^) were suspended in 100 μl PBS and inoculated subcutaneously into the flank of BALB/c athymic nude mice. All mice were supplemented with estrogen pellets. Tumors were measured every other two days after they were visible to the naked eye. Sixteen days after tumor cell transplantation, cholesterol-conjugated miR-30c agmir (1 nmol) in 0.1 ml saline were intratumorally injected into the tumor mass every 3 days for 2 weeks. Adriamycin (1 mg/kg) was administered intravenously three times a week for 2 weeks. Tumor growth was monitored by caliper measurement twice a week. Tumor volume (V) was determined by the length (L) and width (W) according to the following formula: V = (*L* × *W*^2^) /2. Mice were sacrificed, and tumors were removed and weighed 34 days after tumor transplantation.

### Statistical analysis

Statistical analysis was performed using SPSS17.0 statistical software (SPSS Inc, Chicago, IL, USA). Data are presented as the mean ± SD of three independent experiments. Student’s *t*-test was used for comparisons. OS analysis was estimated by the Kaplan–Meier method. The Spearman rank test was used to identify the correlations between miR-30c and REV1 or FANCF. A *p*-value of < 0.05 was considered statistically significant.

## Supplementary information

Supplementary Figure.

Supplementary Methods.

Supplementary Tables.
