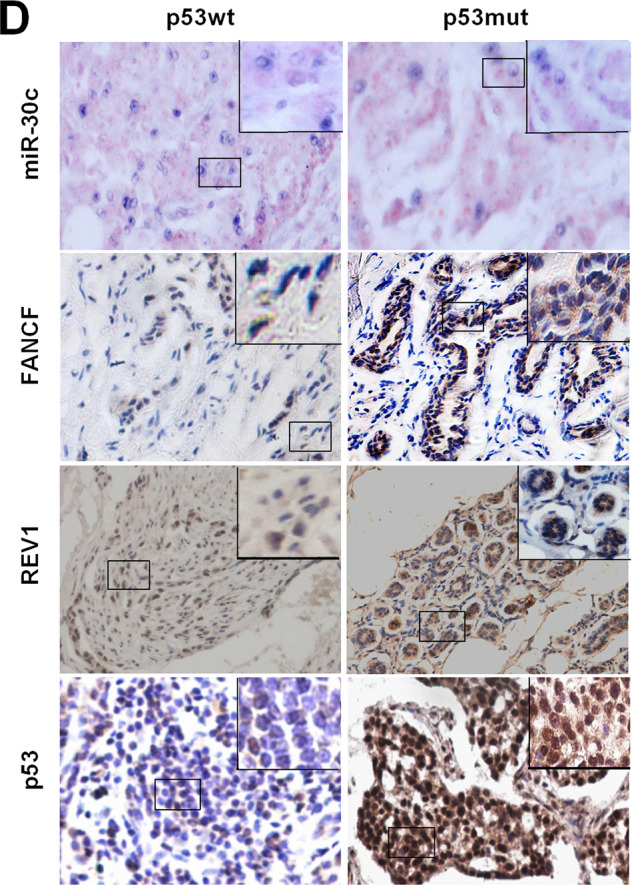# Correction: Intrinsic adriamycin resistance in p53-mutated breast cancer is related to the miR-30c/FANCF/REV1-mediated DNA damage response

**DOI:** 10.1038/s41419-022-04543-z

**Published:** 2022-01-25

**Authors:** Shu Lin, Lifeng Yu, Xinyue Song, Jia Bi, Longyang Jiang, Yan Wang, Miao He, Qinghuan Xiao, Mingli Sun, Olufunmilayo I. Olopade, Lin Zhao, Minjie Wei

**Affiliations:** 1 Department of Pharmacology, School of Pharmacy, China Medical University, No. 77 Puhe Road, Shenyang North New Area, 110122 Shenyang City, Liaoning China; 2 Liaoning Key Laboratory of Molecular Targeted Anti-tumor Drug Development and Evaluation, China Medical University, No.77 Puhe Road, Shenyang North New Area, 110122 Shenyang City, Liaoning China; 3 Section of Hematology/Oncology, Department of Medicine, University of Chicago, Chicago, IL 60637-1463 USA

**Keywords:** Cell biology, Molecular biology

Correction to: *Cell Death Dis* (2019): 10:666 10.1038/s41419-019-1871-z, published online 11 September 2019

The original version of this article unfortunately contained mistakes in Figs. 4d and 6d. The authors apologize for the error. The correct figures can be found below.

**Figure 1:**
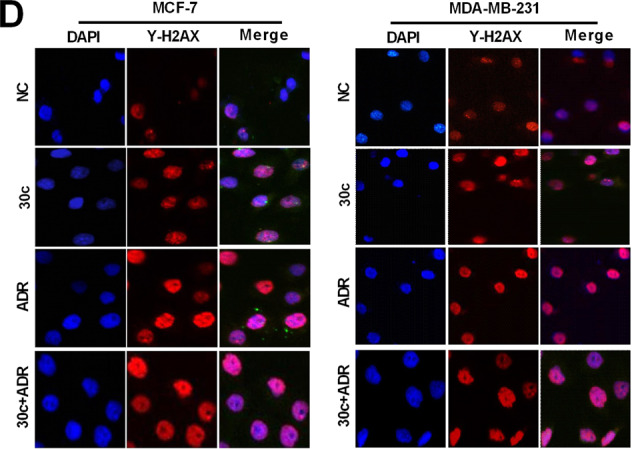

**Figure 2:**