# Supplementary material for: Intrinsic adriamycin resistance in p53-mutated breast cancer is related to the miR-30c/FANCF/REV1-mediated DNA damage response
Source: Cell Death Dis. 2019 Sep 11;10(9):666. doi: 10.1038/s41419-019-1871-z (PMC6739306; doi:10.1038/s41419-019-1871-z)
Supplement: Supplementary file 1 — Supplementary Figure. [file 41419_2019_1871_MOESM1_ESM.doc]

**Supplementary Figures 1-16**

**
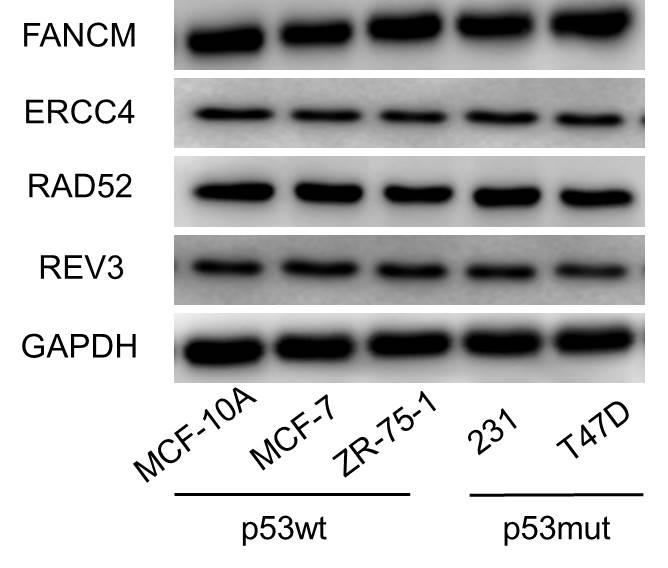
**

**Supplementary Figure S1.** **Western blot analysis of major DNA repair-related proteins in BrCa cell with different p53 status.** The expression of four repair proteins, FANCM, ERCC4, RAD52, and REV3, was not significantly different between p53 wild-type and mutant breast cancer cells.

**
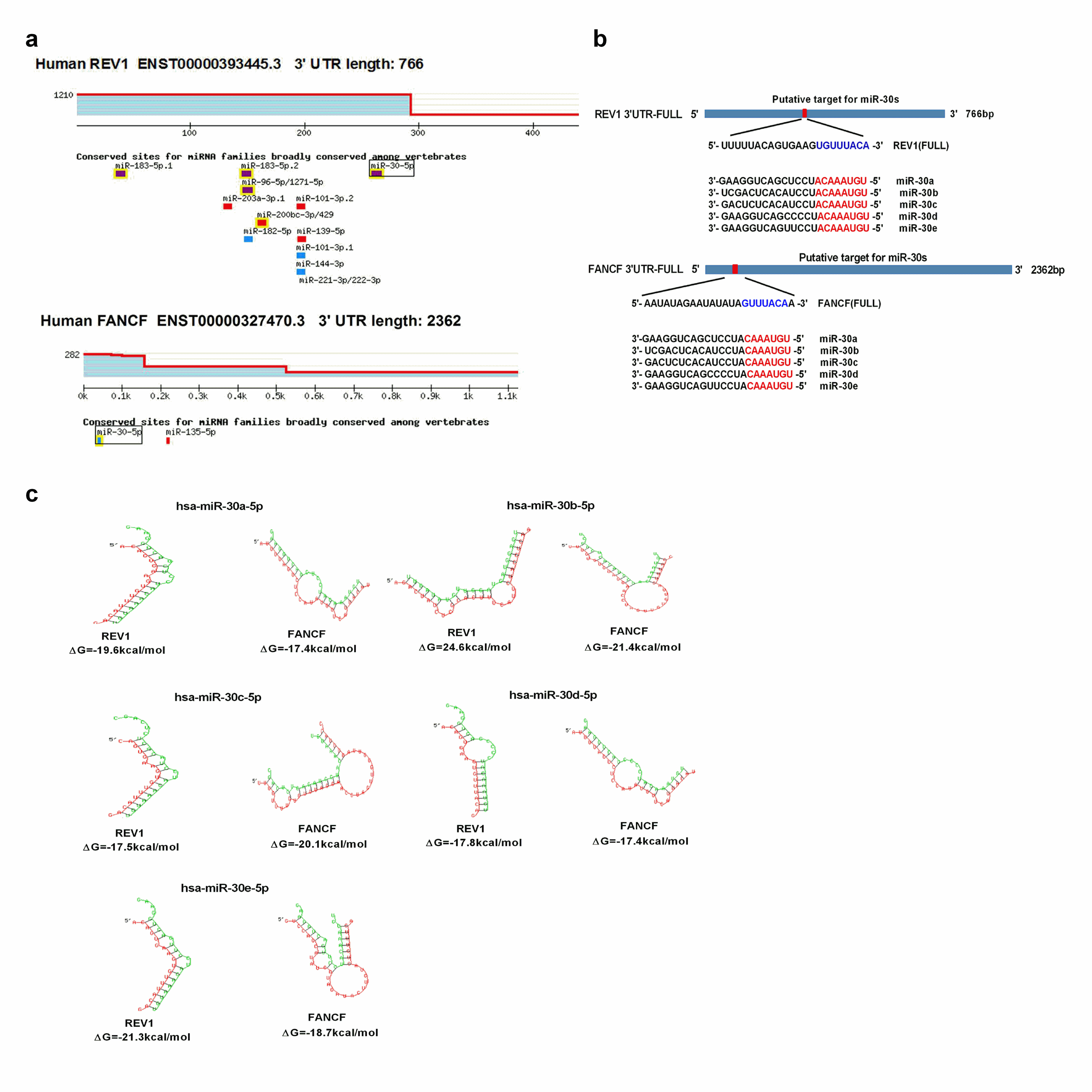
**

**Supplementary Figure S2.** **Bioinformatics predicted the miRNA targeting REV1 and FANCF-3'UTR region. (a)** TargetScan predicted that miR-30 family targets the 3 'UTR of REV1 and FANCF . **(b)**The putative miR-30abcde targeted sequence in the REV1 and FANCF mRNA. **(c)**RNAhybrid predicts one binding site of miR-30abcde in the REV1 and FANCF 3’-UTRs. The binding energy was calculated.


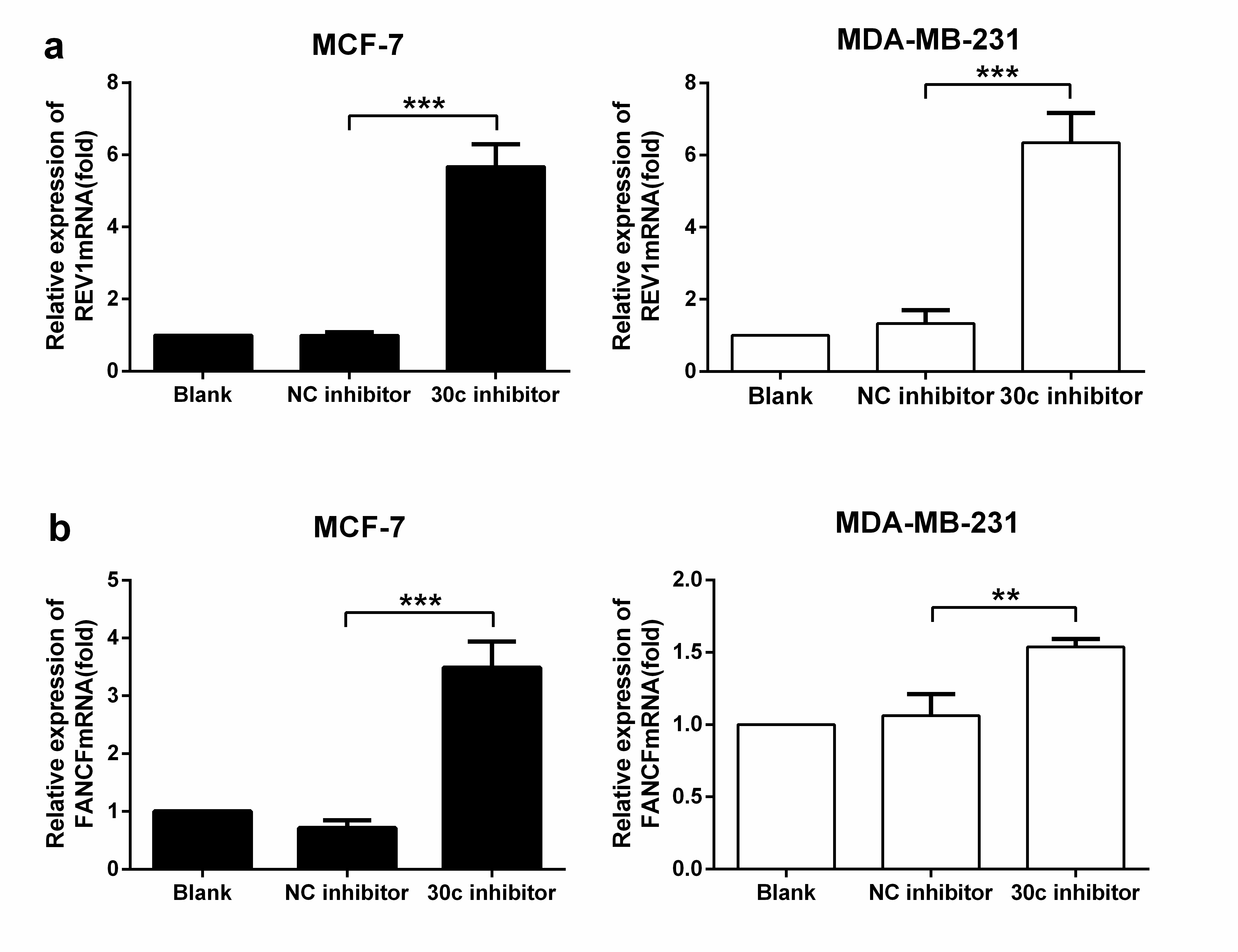


**Supplementary Figure S3.** **Inhibition of endogenous miR-30c by miR-30c inhibitors increased the mRNA of REV1 and FANCF in breast cancer cells.** Cells were transfected with miR-30c inhibitor (20 nM) for 48h and expression levels of REV1mRNA (a) and FANCF mRNA (b) were determined by qRT-PCR analyses. ** p<0.01,*** p<0.001 vs. NC inhibitor group. Data represent the mean±SD (*n*=3, each group) .


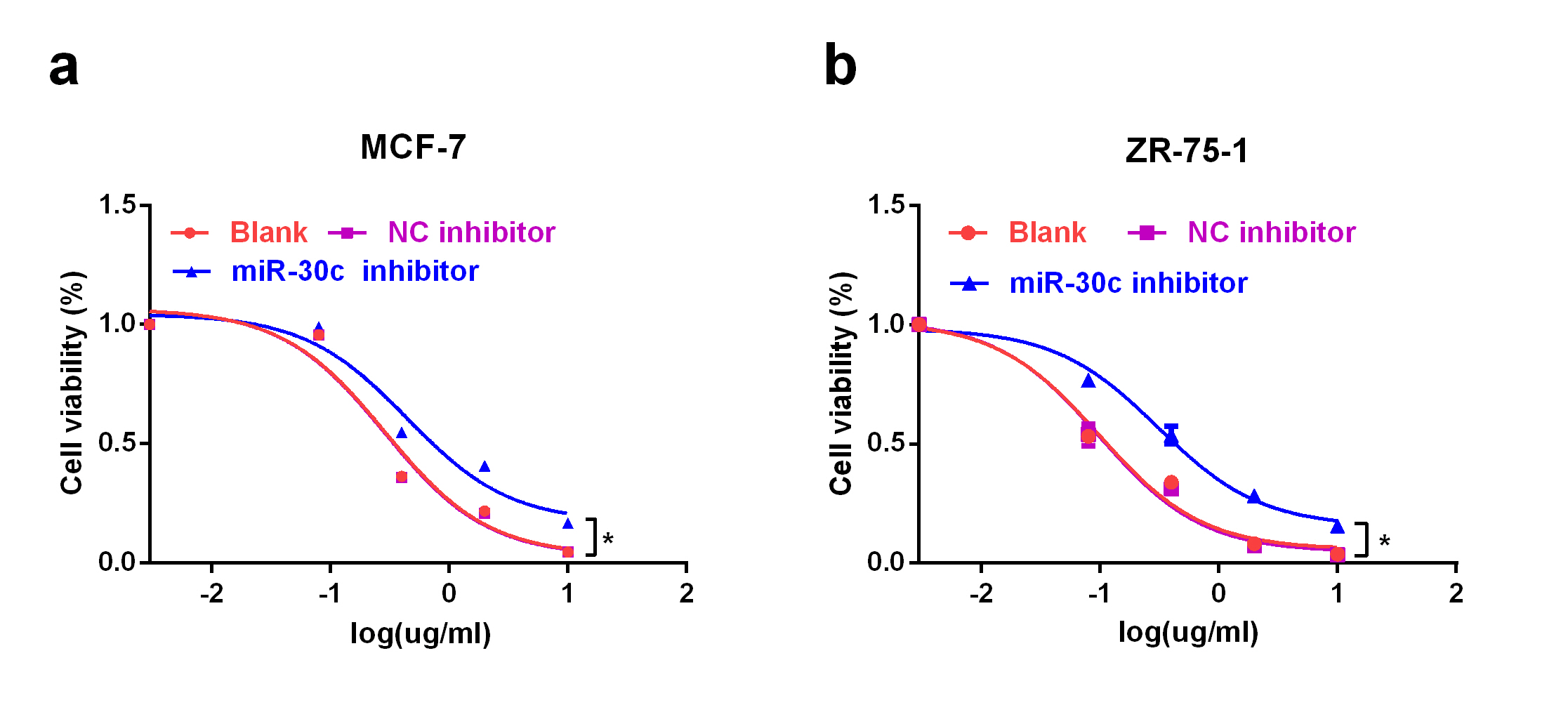


**Supplementary Figure S4. Inhibition of miR-30c significantly decreased chemosensitivity to adriamycin in both MCF-7 and ZR-75-1 cells.** MCF-7(a) and ZR-75-1 (b) cells were transiently transfected with NC inhibitor , miR-30c inhibitor and treated with adriamycin. * p<0.05 vs. NC inhibitor group. Data represent the mean±SD (*n*=3, each group) .


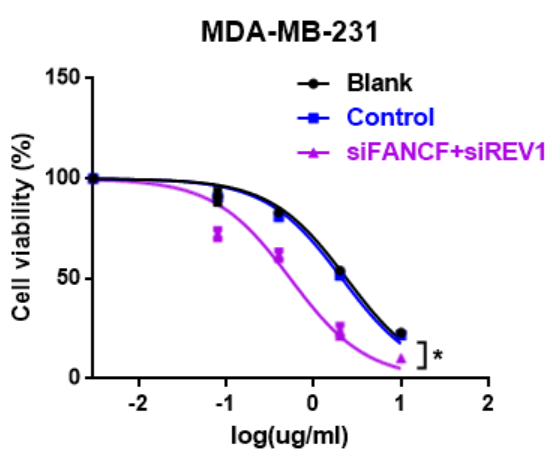


**Supplementary Figure S5.** The relative viability of the mutp53 cell line MDA-MB-231 was detected by CCK-8 assays 48 h after transfection with Control (20nM), FANCFsiRNA+REV1siRNA (20nM). *p<0.05 vs. Control group.


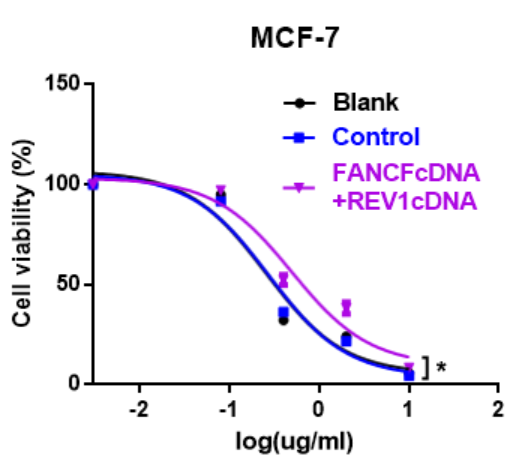


**Supplementary Figure S6.** The relative viability of the wtp53 cell line MCF-7 was detected by CCK-8 assays 48 h after transfection with Control (20nM), FANCFcDNA+REV1cDNA (4μg). *p<0.05 vs. Control group.


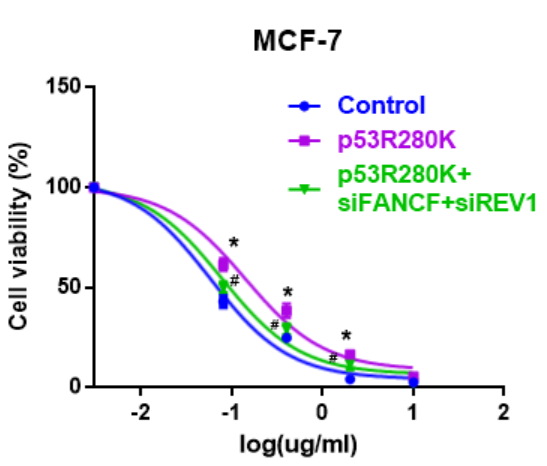


**Supplementary Figure S7.** The wtp53 cell line MCF-7 expressing an empty vector (control) or transduced with mutp53 protein (p53R280K), p53R280K(4μg) +REV1siRNA (20nM)+FANCFsiRNA(20nM) were treated with various ADR concentrations. Cell viability was assessed by CCK-8 assays. *p<0.05 vs. control group, #p<0.05 vs. p53R280K group.

**
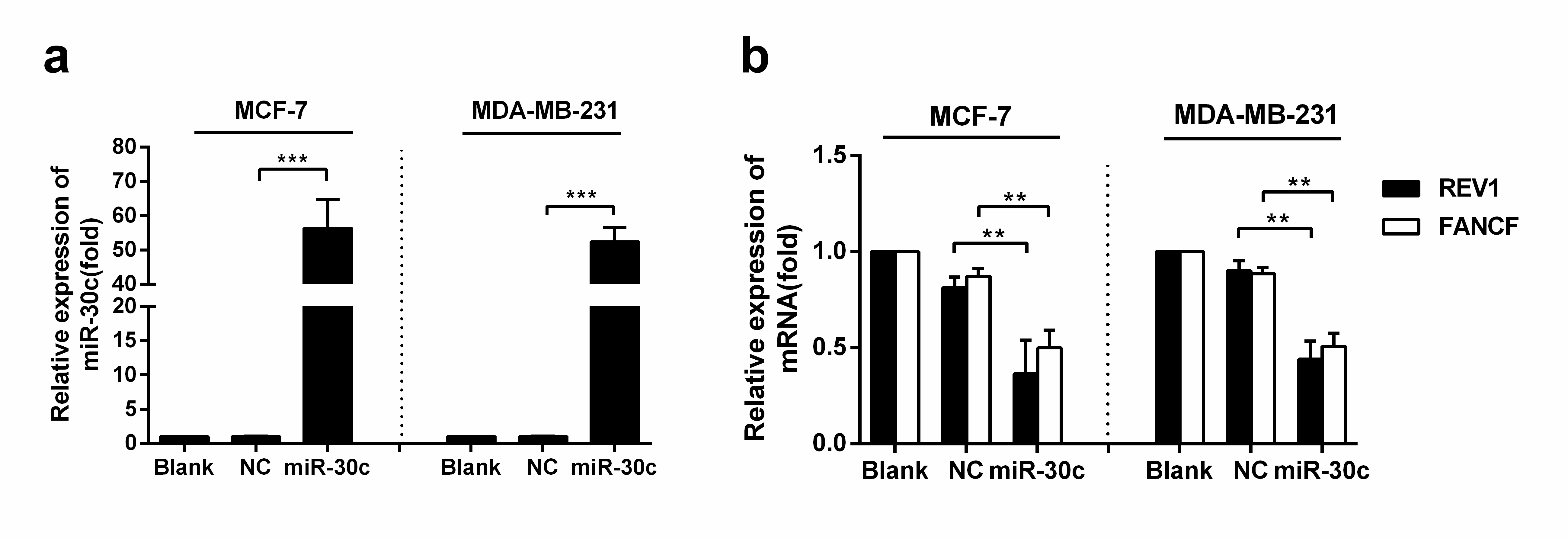
**

**Supplementary Figure S8.** **miR-30c regulated the mRNA expression of REV1, FANCF *in* *vivo.*** (**a**) qRT-PCR showing the relative expression of miR-30c in untreated tumors (Blank), or tumors treated with miR-30c agomir or negative controls (NC). (**b**) qRT-PCR results showing the relative expression of REV1 and FANCF mRNAs in xenograft tumor. Data are showed as mean ±SD. *p＜0.05, **p＜0.01, ***p＜0.001 vs. NC group.


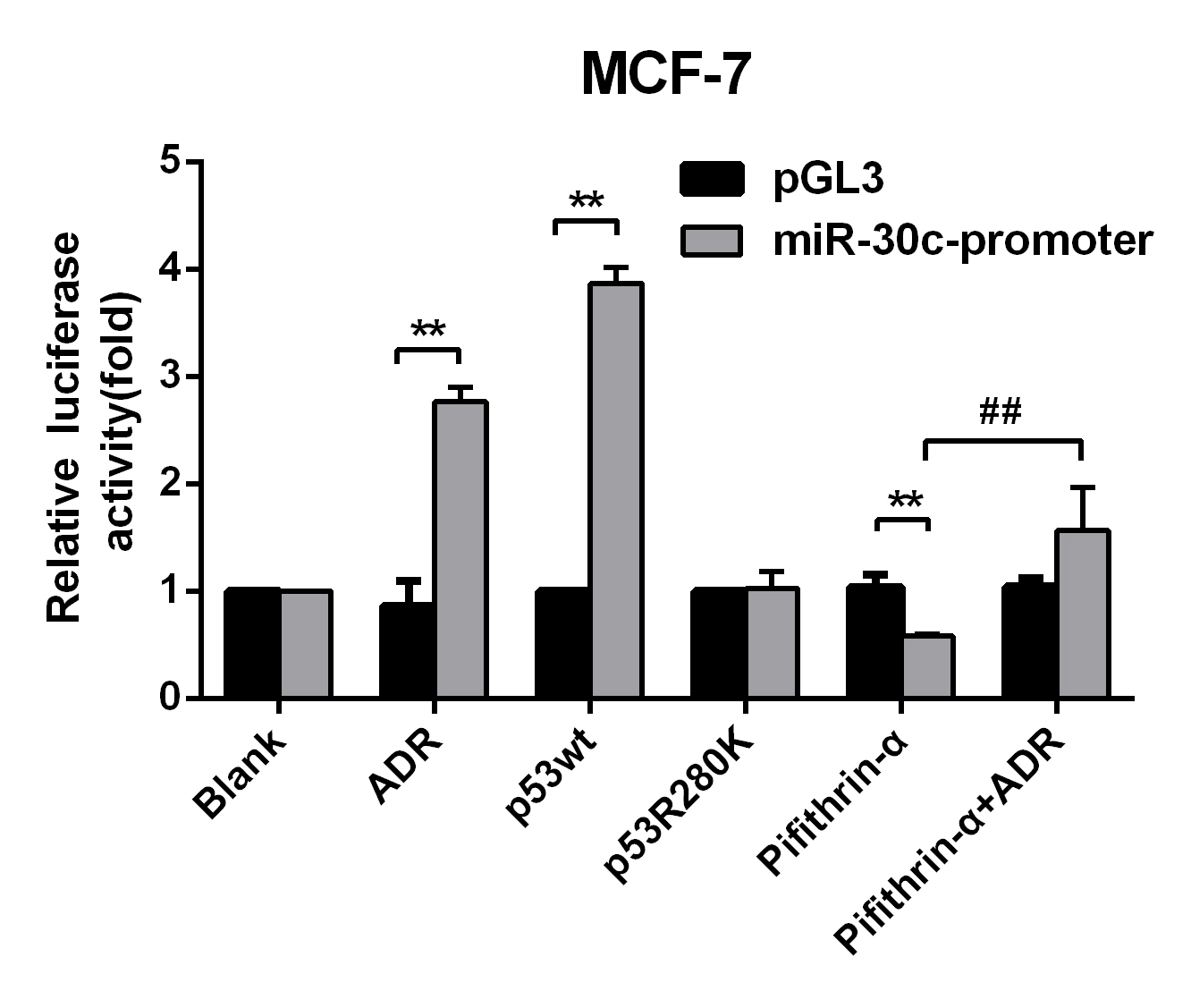


**Supplementary Figure S9.** MCF-7 cells were treated with pcDNA3.1-p53 (wtp53), pcDNA3.1-p53R280K (mutp53), ADR (0.5 µM), pifithrin-α (10 µM), pifithrin-α + ADR and the miR-30c promoter constructs (in the pGL3 vector). Relative luciferase activity was assayed. **p<0.01 vs. PGL3 vector, ^##^p<0.01 vs. pifithrin-α group.

**
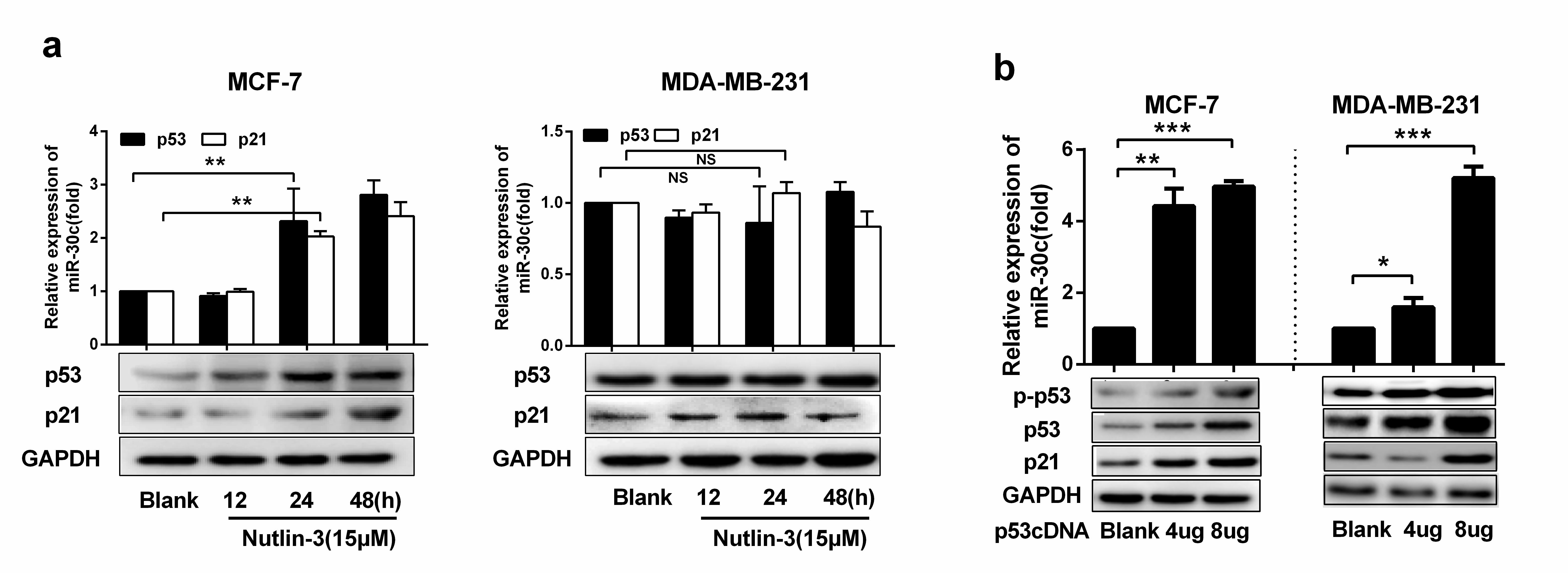
**

**Supplementary Figure S10. The effect of p53 cDNA and nutlin-3 on the expression of miR-30c.** **(a)** MCF-7 and MDA-MB-231 cells were treated with p53 agonist Nutlin-3 (15uM) for 12, 24 and 48h. The expression of mature miR-30c was evaluated by qRT-PCR. The protein expression of p53 and p21 was examined by Western blot (shown below). **(b)** MCF-7 and MDA-MB-231 cells were treated with pcDNA3.1-p53 plasmid or pcDNA3.1 control vector. The expression of mature miR-30c was evaluated by qRT-PCR. The protein expression of p-p53,p53 and p21 was examined by Western blot (shown below). *p<0.05,** p<0.01,*** p<0.001 vs. Blank group. Data represent the mean±SD (*n*=3, each group) .

**
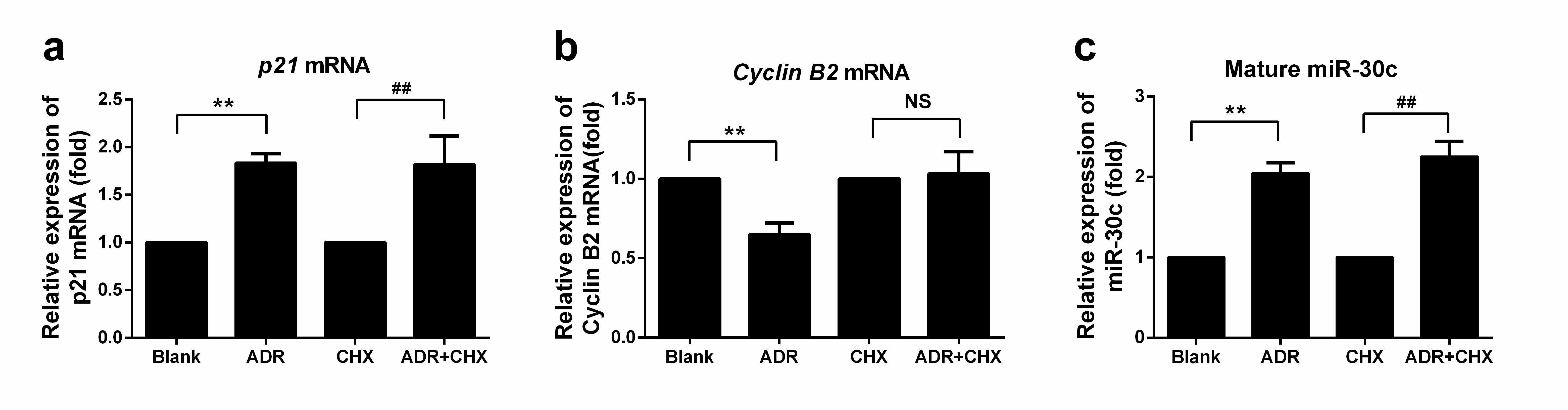
**

**Supplementary Figure S11. p53 transcriptionally upregulates miR-30c**. After MCF-7 cells were treated with cycloheximide (CHX) for 6 h, ADR treated MCF-7 cells for 24h. MRNA induction of the direct target gene p21CIP1/WAF1 (**a**) and downregulation of indirectly controlled Cyclin B2 mRNA (**b**). (**c**) miR-30c induction was investigated by RT-PCR with or without prior CHX treatment. The fold regulations of the transcripts relative to the respective controls are shown. *p＜0.05, * *p＜0.01 vs Blank group,##p＜0.01 vs. CHX group.


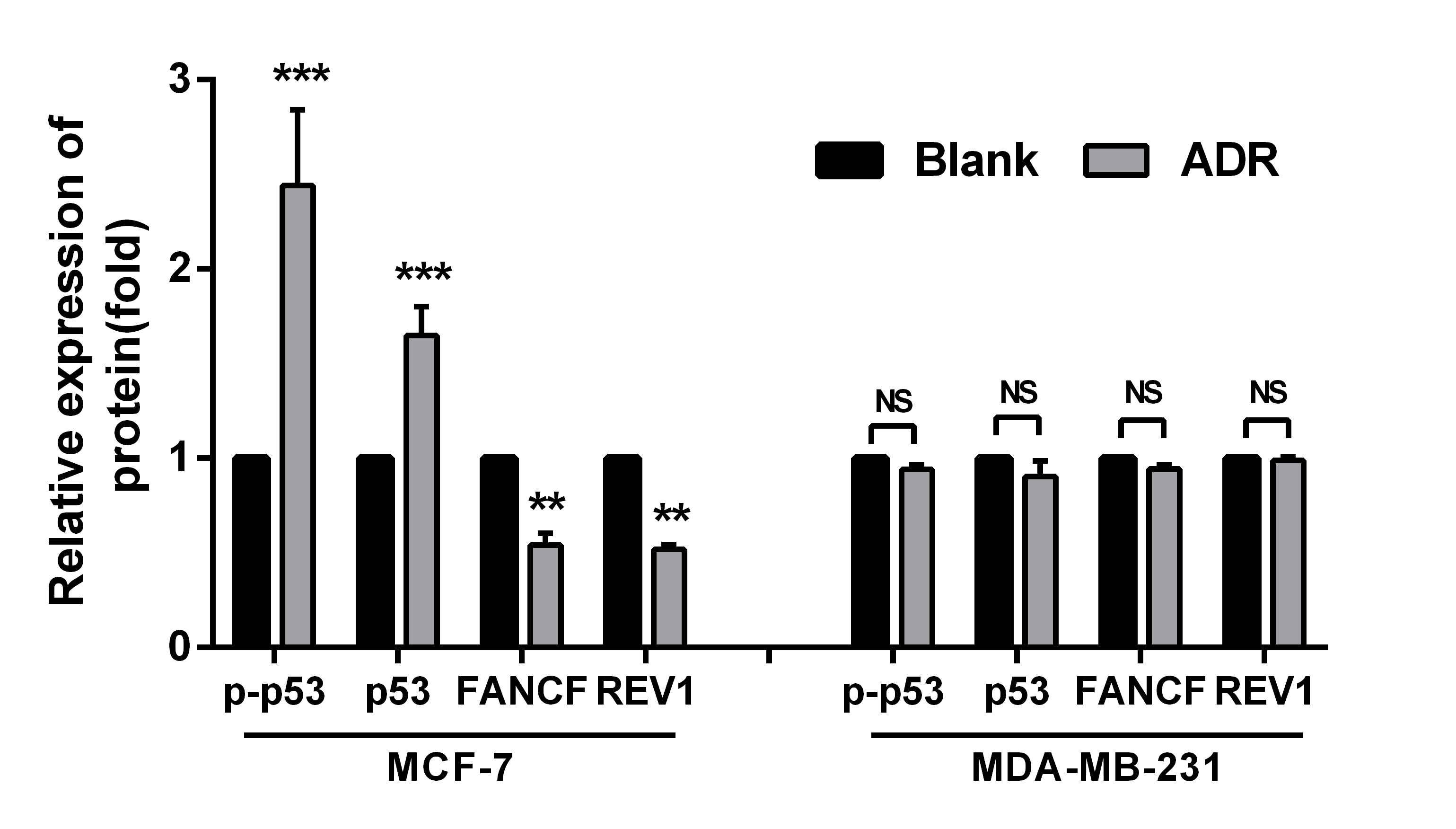


**Supplementary Figure S12.** The statistical results of p-p53, p53, FANCF and REV1 expression in ADR-treated MCF-7 and MDA-MB-231 xenografts. **p<0.01, ***p<0.001 vs. Blank group. NS indicates no significant difference. Data represent the mean ± SD (*n*=3, each group).


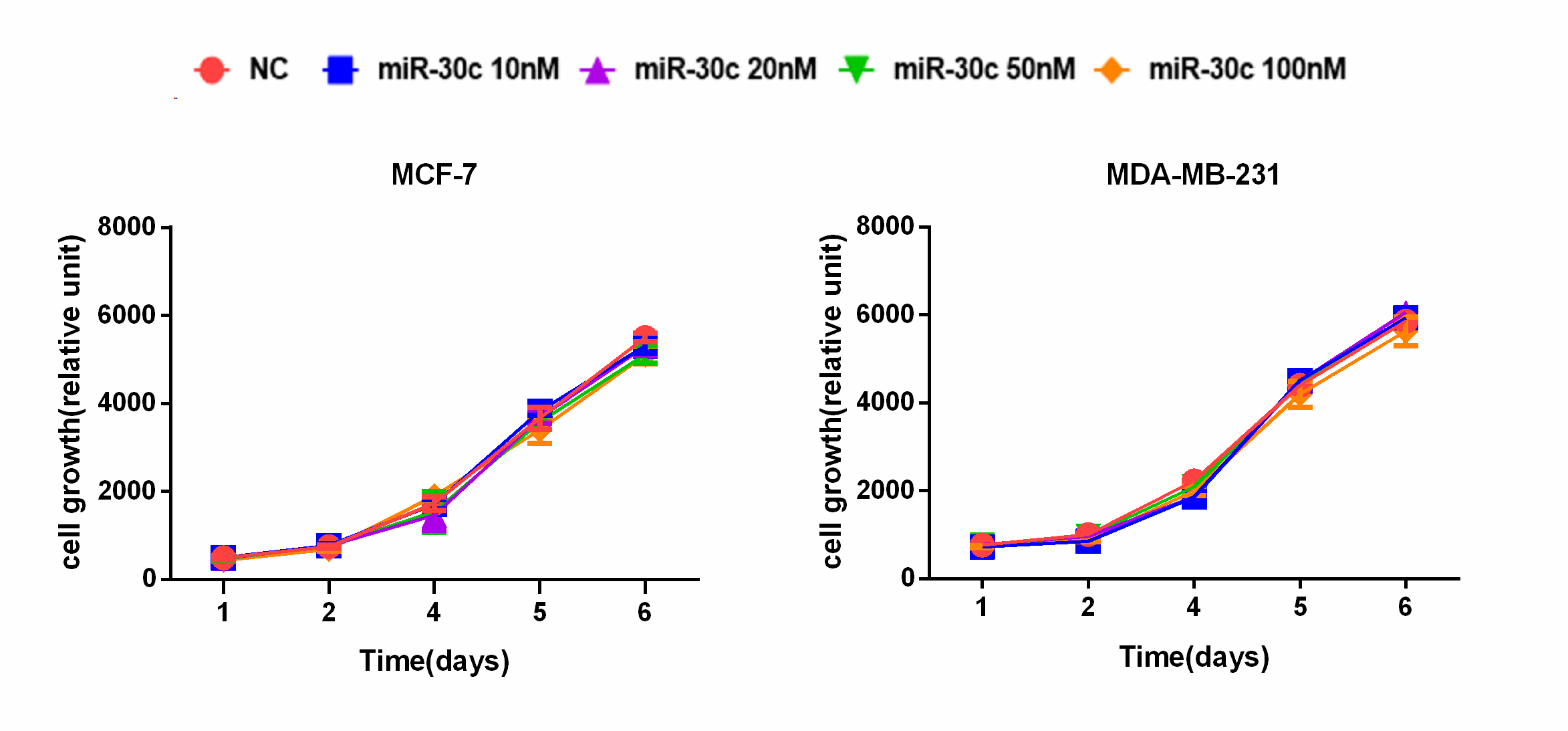


**Supplementary Figure S13.** The growth rate of the MCF-7 and MDA-MB-231 cells were detected 1,2,4,5,6 days after transfection with NC(the scrambled RNA mimic), miR-30c mimic (10 nM), miR-30c mimic (20 nM), miR-30c mimic (50 nM) and miR-30c mimic (100 nM). No significant effect of transient transfection of different concentration miR-30c on the growth rate of MCF-7 and MDA-MD-231 cells, compared to the NC group.


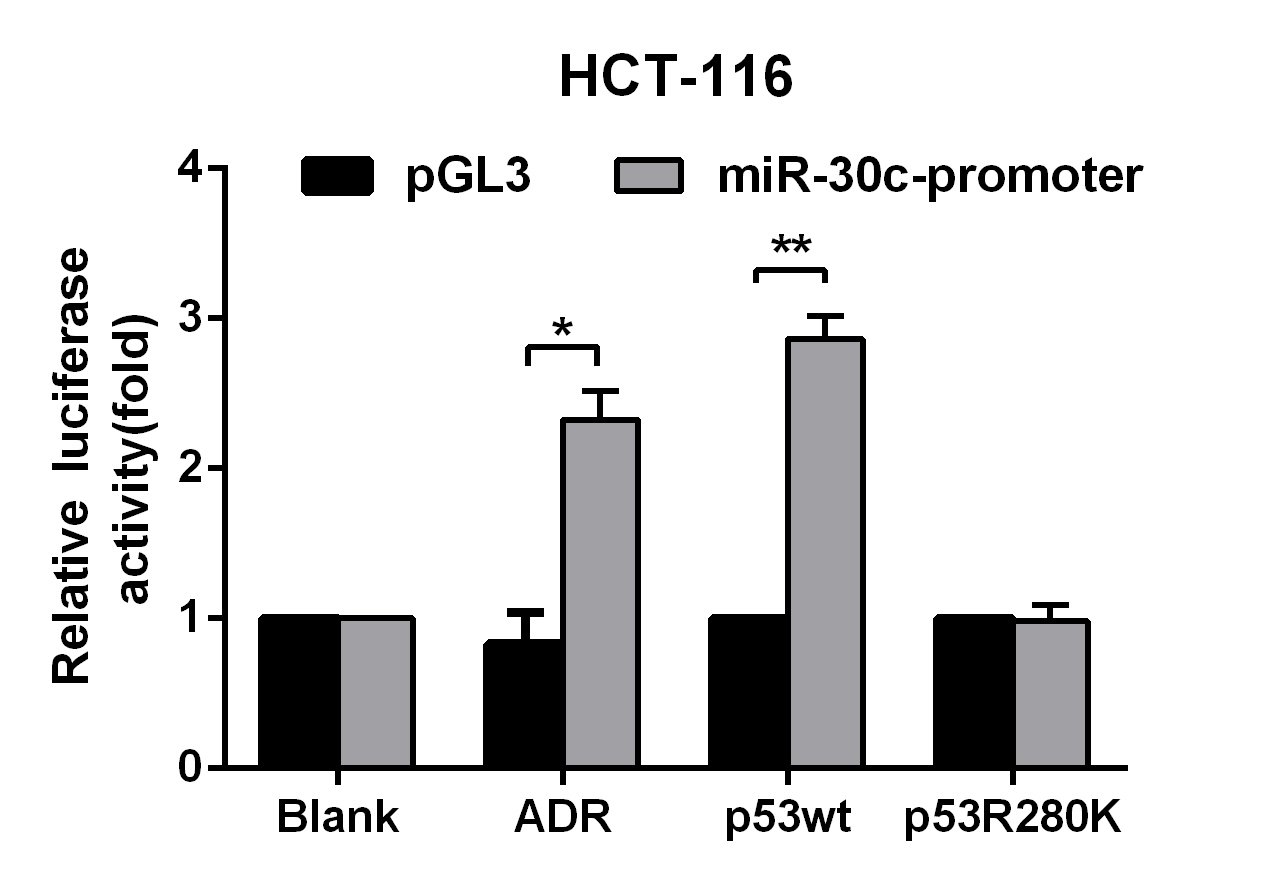

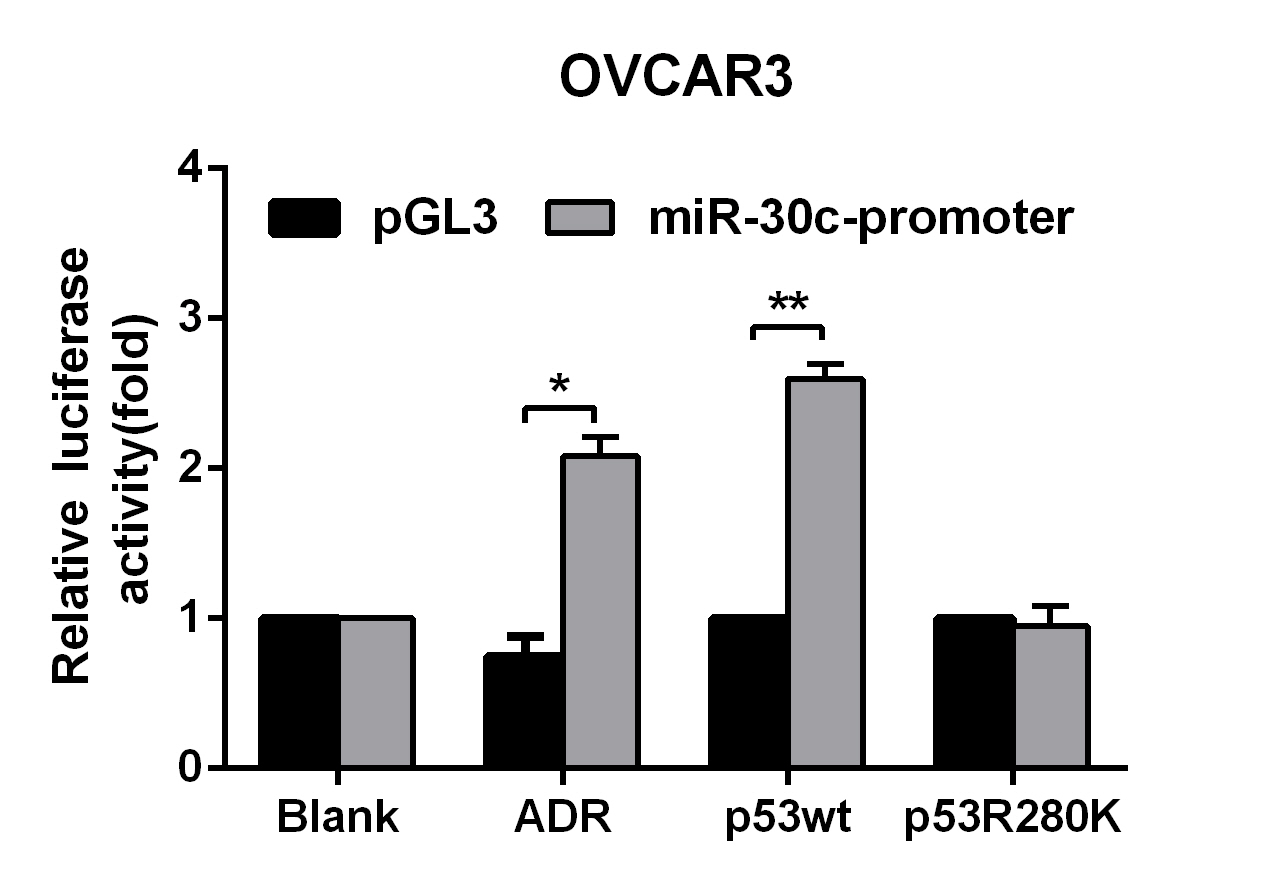


**Supplementary Figure S14.** HCT-116 and OVCAR3 cells were treated with pcDNA3.1-p53 (wtp53), pcDNA3.1-p53R280K (mutp53), ADR (0.5 µM) and the miR-30c promoter constructs (in the pGL3 vector). Relative luciferase activity was assayed. *p<0.05, **p<0.01 vs. PGL3 vector.


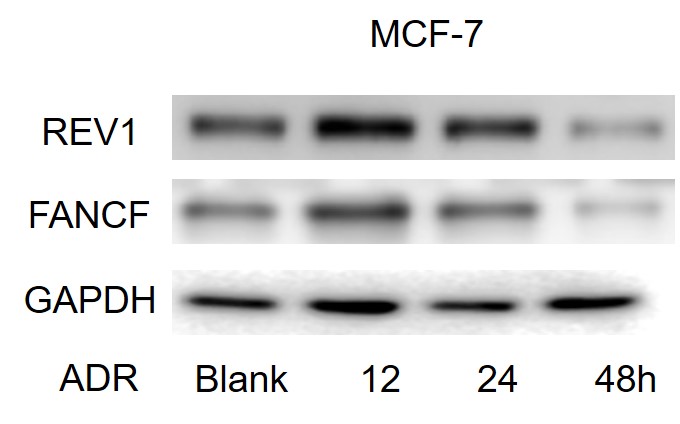


**Supplementary Figure S15.** MCF-7 cells were treated with ADR (0.5 µM) for 0,12,24,48 h. The cells were lysed for Western blotting and probed with the indicated antibodies.


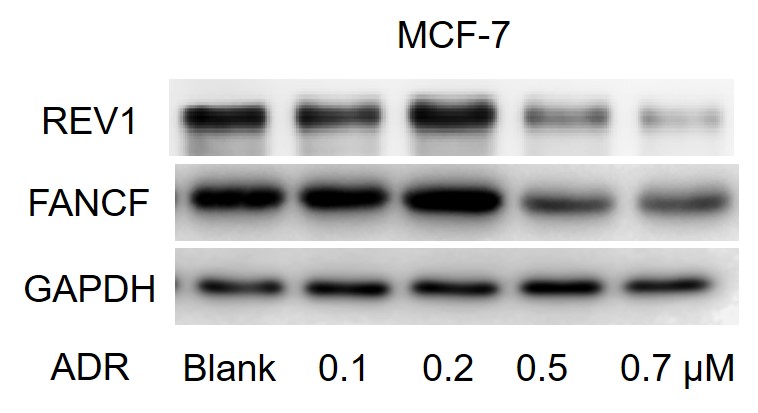


**Supplementary Figure S16.** MCF-7 cells were treated with ADR (0.1, 0.2, 0.5, 0.7 µM) for 48 h. The cells were lysed for Western blotting and probed with the indicated antibodies.
