# Supplementary material for: Intrinsic adriamycin resistance in p53-mutated breast cancer is related to the miR-30c/FANCF/REV1-mediated DNA damage response
Source: Cell Death Dis. 2019 Sep 11;10(9):666. doi: 10.1038/s41419-019-1871-z (PMC6739306; doi:10.1038/s41419-019-1871-z)
Supplement: Supplementary file 2 — Supplementary Methods. [file 41419_2019_1871_MOESM2_ESM.doc]

**Supplementary Materials and Methods**

**Quantitative real-time PCR**

Total RNA was isolated from tumor cells using TRIZOl reagents (Invitrogen, CA , USA) according to the manufacture's protocol. qRT-PCR were performed using SYBR Premix ExTaq™ II kit (Takara). The primers are listed in Supplementary Table S2. Relative expression of miR-30c was normalized to U6 expression and REV1 and FANCF was to GAPDH. The fold change for each RNA relative to the control was calculated using the 2^-△△Ct^ method.

**Western blot analysis**

Cells were collected and homogenized on ice in lysis buffer. Proteins were separated by 10% SDS-PAGE electrophoresis, and transferred onto polyvinylidene fluoride membranes. Membranes were blocked with 1% BSA for 1 h, and incubated with primary antibodies at 4°C overnight, followed by incubation with secondary antibodies for 1 h at room temperature. The antibodies used are described in Supplementary Table S2. Bands were visualized with a chemiluminescent detecting system (Amersham, Freiburg, Germany). The intensity of the western blot bands was analyzed by ImageJ software (http://rsb.info.nih.gov/ij).

**Luciferase activity assay**

The human REV1-3’-UTR (766 bp) and FANCF-3’-UTR (2362 bp) was amplified by PCR and cloned into the XbaI site of the pGL3 vector (Promega). Mutations in the miRNA-binding site were generated using PCR-based mutagenesis (Takara, Dalian, China). To generate miR-30c promoter plasmids, the miR-30c promoter regions (-1832~+150bp) were amplified by PCR and cloned to the pGL3-Basic vector (Promega, WI, USA), respectively. The primers used are listed in Supplementary Table S1.

To study the effect of miR-30c on FANCF and REV1 expression via targeting the 3′-UTRs of FANCF and REV1, Luciferase reporter vectors (control) or vectors containing wild type (pGL3-REV1-3'UTR-Full, pGL3-FANCF-3'UTR-Full) or mutated 3'-UTRs (pGL3-REV1-3'UTR-Mut, pGL3-FANCF-3′UTR-Mut) of REV1 and FANCF mRNAs were co-transfected into 293T cells with miR-30c, using Lipofectamine 2000. To study the direct binding of p53 to the promoter region of miR-30c, the miR-30c-promoter reporter constructs were cotransfected with p53cDNAwt, or p53cDNA-R280K into HEK-293T cells using Lipofectamine 2000. After transfection for 48 h, luciferase activity was detected using the Dual Luciferase Reporter Gene Assay kit (Promega). Relative luciferase activity normalized to the control.

**MiR-30c *in situ* hybridization**

Paraffin sections were mounted on Super frost +glass slides and deparaffinized. The slides were then treated with proteinase-K 10 μg/ml at 37℃ for 10min, pre-hybridizd in Exiqon hybridization buffer (Exiqon, Vedbæk, Denmark) at 37℃ for 2h, hybridized with 50nM miRNA-30c probe and washed stringently with 5× SSC, 1 × SSC and 0.2 × SSC buffers at 37℃ for 30 min. DIG blocking reagent (Roche, Mannheim, Germany) was added for 1h at 37 °C in maleic acid buffer with 2% sheep serum. After which, alkaline phosphatase-conjugated anti-digoxigenin was added at 4℃ for 12h (1:500 in Roche blocking reagent). 4-nitroblue tetrazolium (NBT) and 5-brom-4-chloro-3’-Indolylphosphate (BCIP) substrate (Roche) were used for enzymatic development to form dark-blue NBT-formazan precipitates at 37℃ for 60 min. The sections were lightly counterstained with nuclear fast red (Vector Laboratories, Burlingname, CA) at 25℃ for 1 min and mounted. The expression of miR-30c was detected, using digoxin-labelled locked nucleic acid-modified RNA probes against the full length mature miR-30c sequence. MiR-30c probe sequences are listed in Table S1.

**Immunohistochemistry**

For immunohistochemistry, slides were deparaffinized and rehydrated. Antigen retrieval was performed by a pressure cooker for 10min in 0.01M citrate buffer (pH 6.0), followed by treatment with 3% hydrogen peroxide for 5min. Next, slides were blocked in sheep serum for 30 min, and then incubated with antibodies specific for REV1（Rabbit Polyclonal 1:50 dilution, novus）and FANCF (Rabbit Polyclonal 1:200 dilution, Bioss) overnight at 4°C. Immunostaining was performed using DAB according to the manufacturer’s instructions. The slides were mounted and the images were captured and analyzed by a fluorescence microscope.

**Cell proliferation**

Cells were transfected with 4ug p53R280K, 4ug p53cDNA, 20 nM miR-30c mimics or 20 nM miR-30c inhibitor for 24 h. Cells were then seeded onto 96-well plates at a density of 4x10^3^ cells/well. After culture for 24 h, cells were treated with serial dilutions of ADR for 48 h, followed by treatment with CCK-8 (Dojindo Molecular Technologies Inc., Japan) for 1 h. The absorbance at 450 nm was measured using a multi-mode reader (LD942, Beijing, China).

**Animal study**

To establish breast tumor xenograft model, MCF-7 and MDA-MB-231cells (5×10^6^) were suspended in 100 μl PBS and inoculated subcutaneously into the flank of BALB/c athymic nude mice. All mice were supplemented with estrogen pellets. Tumors were measured every other two days after they were visible to the naked eye. Sixteen days after tumor cell transplantation, cholesterol-conjugated miR-30c agmir (1 nmol) in 0.1 ml saline were intratumorally injected into the tumor mass every 3 days for 2 weeks. Adriamycin (1 mg/kg) was administered intravenously three times a week for 2 weeks. Tumor growth was monitored by caliper measurement twice a week. Tumor volume (V) was determined by the length (L) and width (W) according to the following formula: V = (*L*×*W*^2^) /2. Mice were sacrificed, and tumors were removed and weighed 34 days after tumor transplantation. All animal work was done in accordance with a protocol approved by the Animal Center of and Animal Ethics Committee of China Medical University.
