# Supplementary material for: Intrinsic adriamycin resistance in p53-mutated breast cancer is related to the miR-30c/FANCF/REV1-mediated DNA damage response
Source: Cell Death Dis. 2019 Sep 11;10(9):666. doi: 10.1038/s41419-019-1871-z (PMC6739306; doi:10.1038/s41419-019-1871-z)
Supplement: Supplementary file 3 — Supplementary Tables. [file 41419_2019_1871_MOESM3_ESM.doc]

**Supplementary Tables**

**Supplementary Table 1. The sequences for primers used in the study**

| **Name** | **Sequences** |
| --- | --- |
| *Primers for qRT-PCR* | |
| REV1-F  REV1-R  FANCF-F  FANCF-R | 5′- GTCCAGCAAGCAGAGTCACA -3′  5′-GGAAGGGCAGCAAATACCTC-3′  5′-TTTTTGTGTTTGTTGGAGAATTGGGTTTTT-3′  5′-ATACACCACAAACCACCAACAAACAAAACA-3′ |
| GAPDH-F | 5′- TTGCCCTCAACGACCCTTT -3′ |
| GAPDH-R | 5′- TCCTCTTGTGCTCTTGCTGG -3′ |
| *Primers for miR-30c promoter* | |
| primer -F | 5’- CCGCTCGAGAGGGAGGTCGCATTCTGT -3’ |
| primer -R | 5'- CCGCTCGAGATTTCCTTGCTGGCTTGA -3’ |
| *Primers for CHIP* | |
| primer 1-F  primer 1-R  primer 2-F  primer 2-R | 5’- AGCATAATCATATTGAGGGT -3’  5’- GTATCCTTTGTTTGCCTGA -3’  5’- CCTCTAACTGTCTGCCTGT -3’  5’- TGCCCTCAACTGTGCTTC -3’ |
| *Primers for miR-30c ISH* | 5’- Dig-GCTGAGAGTGTAGGATGLTTTACLAL-Dig-3’ |

Abbreviations: qRT-PCR, Quantitative real-time PCR; CHIP, Chromatin Immunoprecipitation;ISH, in situ hybridization;F, forward primer; R, reverse primer.

**Supplementary Table 2.** Antibodies used for IHC and WB

| **Antibody** | **Company/Provider** |
| --- | --- |
| anti-human γ-H2AX | Abcam plc, Cambridge, UK |
| p53 rabbit IgG | Cell Signaling Technology, MA, USA |
| goat anti-mouse IgG | Invitrogen, CA , USA |
| goat anti-rabbit IgG | Invitrogen, CA , USA |
| anti-human REV1(IHC) | Novus Biologicals,USA |
| anti-human FANCF | Abcam plc, Cambridge, UK |
| anti-human REV1 | Abcam plc, Cambridge, UK |
| anti-human FANCD2 | Abcam plc, Cambridge, UK |
| anti-GAPDH | Santa Cruz Biotechnology, CA, USA |
| goat anti-rabbit IgG-HRP | Santa Cruz Biotechnology, CA,USA |
| goat anti-mouse IgG-HRP | Santa Cruz Biotechnology, CA,USA |
